# Evidence for divergent patterns of local selection driving venom variation in Mojave Rattlesnakes (*Crotalus scutulatus*)

**DOI:** 10.1038/s41598-018-35810-9

**Published:** 2018-12-04

**Authors:** Jason L. Strickland, Cara F. Smith, Andrew J. Mason, Drew R. Schield, Miguel Borja, Gamaliel Castañeda-Gaytán, Carol L. Spencer, Lydia L. Smith, Ann Trápaga, Nassima M. Bouzid, Gustavo Campillo-García, Oscar A. Flores-Villela, Daniel Antonio-Rangel, Stephen P. Mackessy, Todd A. Castoe, Darin R. Rokyta, Christopher L. Parkinson

**Affiliations:** 1 Department of Biology, University of Central Florida, 4110 Libra Drive, Orlando, FL 32816 USA; 2 School of Biological Sciences, University of Northern Colorado, 501 20th Street, Greeley, CO 80639 USA; 3 Department of Biological Sciences, Clemson University, 190 Collings St., Clemson, SC 29634 USA; 4 Department of Biology, University of Texas at Arlington, 501 S. Nedderman Drive, Arlington, TX 76010 USA; 5 Facultad de Ciencias Biológicas, Universidad Juárez del Estado de Durango, Av. Universidad s/n. Fracc. Filadelfia, C.P. 35070 Gómez Palacio, Dgo. Mexico; 6 Museum of Vertebrate Zoology, University of California, 3101 Valley Life Sciences Building, Berkeley, CA 94720 USA; 7 Department of Biology and Burke Museum of Natural History and Culture, University of Washington, Box 351800, Seattle, WA 98195 USA; 8 Museo de Zoología, Department of Evolutionary Biology, Faculta de Ciencias, Universidad Nacional Autónoma de México, External Circuit of Ciudad Universitaria, México City, Mexico; 9 Instituto Politécnico Nacional, Escuela Nacional de Ciencias Biológicas, Laboratorio de Cordados Terrestres, Colección Herpetológica, Del. Miguel Hidalgo, México City, Mexico; 10 Department of Biological Science, Florida State University, Tallahassee, Florida 32306 USA; 11 Present Address: Department of Biological Sciences, Clemson Univeristy, 190 Collings St., Clemson, SC 29634 USA; 12 Present Address: Department of Biological Sciences & Department of Forestry and Environmental Conservation, Clemson University, 190 Collings St., Clemson, SC 29634 USA

## Abstract

Snake venoms represent an enriched system for investigating the evolutionary processes that lead to complex and dynamic trophic adaptations. It has long been hypothesized that natural selection may drive geographic variation in venom composition, yet previous studies have lacked the population genetic context to examine these patterns. We leverage range-wide sampling of Mojave Rattlesnakes (*Crotalus scutulatus*) and use a combination of venom, morphological, phylogenetic, population genetic, and environmental data to characterize the striking dichotomy of neurotoxic (Type A) and hemorrhagic (Type B) venoms throughout the range of this species. We find that three of the four previously identified major lineages within *C*. *scutulatus* possess a combination of Type A, Type B, and a ‘mixed’ Type A + B venom phenotypes, and that fixation of the two main venom phenotypes occurs on a more fine geographic scale than previously appreciated. We also find that Type A + B individuals occur in regions of inferred introgression, and that this mixed phenotype is comparatively rare. Our results support strong directional local selection leading to fixation of alternative venom phenotypes on a fine geographic scale, and are inconsistent with balancing selection to maintain both phenotypes within a single population. Our comparisons to biotic and abiotic factors further indicate that venom phenotype correlates with fang morphology and climatic variables. We hypothesize that links to fang morphology may be indicative of co-evolution of venom and other trophic adaptations, and that climatic variables may be linked to prey distributions and/or physiology, which in turn impose selection pressures on snake venoms.

## Introduction

Studying the context and geographic scale of local adaptation is critical to understanding the processes that shape the evolution of adaptive traits in natural populations^1–3^. Snake venom represents an excellent model for studying variation across populations as a result of stochastic and deterministic evolutionary processes. For example, strong selection is expected to act on snake venom due to its important ecological role in feeding and defense^4–7^, and because it is composed of numerous components that together manifest as distinct venom phenotypes between populations and species^4,8^. Additional dimensions involved in venom evolution are the physiological factors that must co-evolve with venom to allow for its production and prevent auto-toxicity^9–12^. Thus, snake venom provides opportunity to discern the causes of variation that have direct consequences for individual fitness at multiple scales.

There are several intriguing examples of phenotypic polymorphism in venom within rattlesnake species^5,13,14^, with the most well-studied example existing in Mojave Rattlesnakes (*Crotalus scutulatus*). In *C*. *scutulatus*, venom composition among individuals generally takes one of two forms, either a highly neurotoxic ‘Type A’ venom, or a hemorrhagic ‘Type B’ venom^4,15–20^. The venom of Type A individuals is dominated by a neurotoxin formed from a heterodimeric phospholipase A_2_ (PLA_2_) called Mojave Toxin (MTX) and has little snake venom metalloproteinase (SVMP) activity. Type B individuals lack MTX and tend to have high expression of SVMPs in their venom. Individuals possessing both phenotypes (i.e., Type A + B individuals) have also been found, though less frequently than strictly Type A and Type B individuals^21,22^.

**Figure 1:**
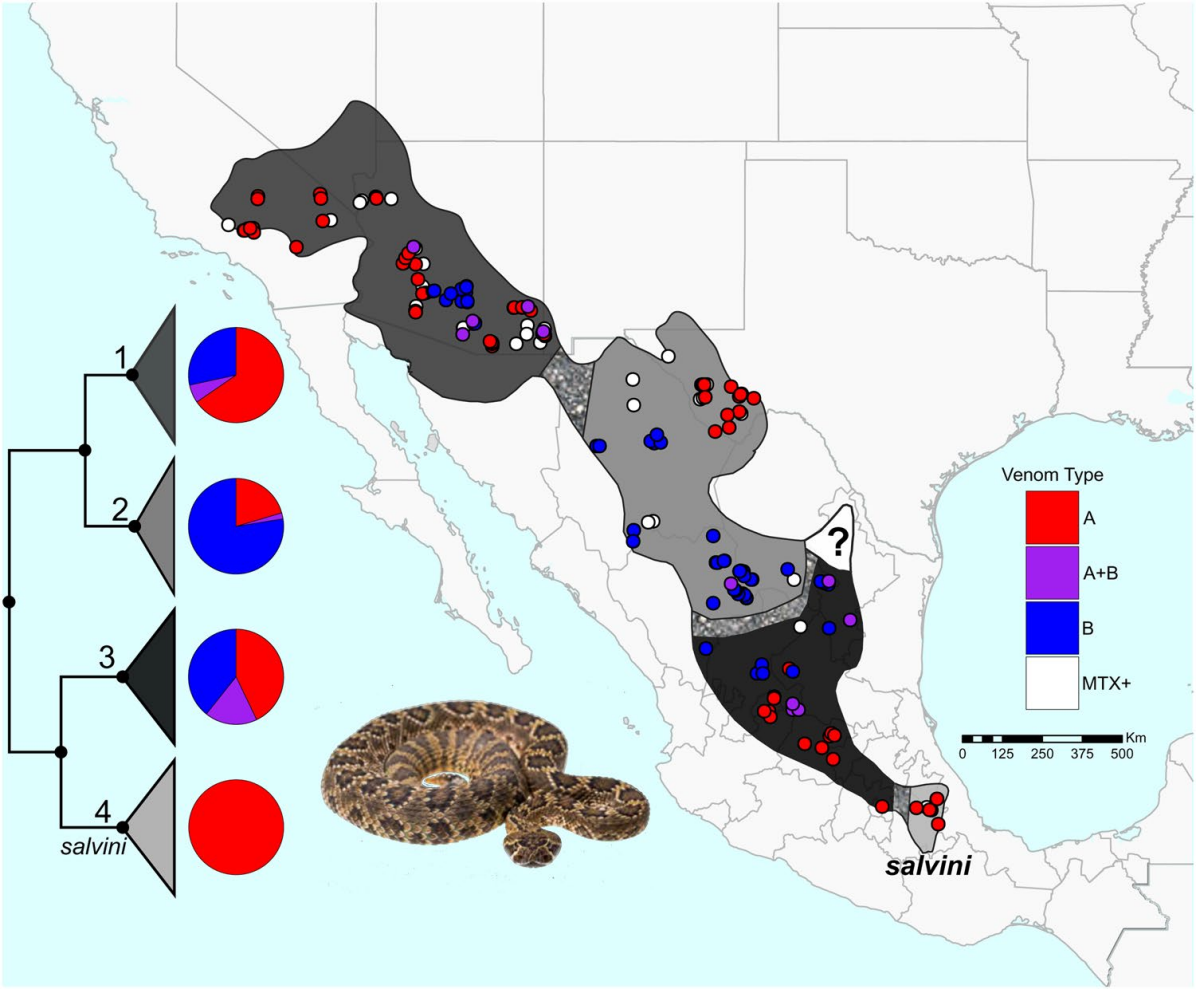
Distribution and sampling of Mojave Rattlesnakes collected from throughout their range. Red, purple, and blue represent Type A, Type A + B, and Type B venom, respectively, in the pie charts and the sampling points. White points are individuals that were positive for Mojave Toxin but we could not distinguish between Type A and Type A + B. Mottling in the distribution are areas of gene flow between lineages. Pie charts represent the proportion of each venom type collected from each lineage based on samples with venom. Cladogram in lower left of the four lineages from Fig. 3 corresponding to those in Schield *et al*.^38^ numbered: 1 - Sonoran lineage, 2 - Chihuahuan lineage, 3 - Central Mexican Plateau lineage, 4 - *salvini* lineage, ?-not sampled/unknown lineage. Inset photo: *Crotalus scutulatus* by Travis Fisher.

Mojave Rattlesnakes are distributed in the deserts of North America, the Central Mexican Plateau, and the volcanic lowlands of south-central Mexico (Fig. 1). While previous proteomic^16,19,23,24^ and transcriptomic^25^ studies have shown that Mojave Rattlesnakes possess high intraspecific venom variation, venom composition has not been well characterized for the majority of the geographic distribution of these snakes. Type A, Type B, and Type A + B phenotypes are known from several distinct locations throughout the species distribution, and the spatial assortment of these phenotypes suggests that they are geographically structured^15,19,21–23,26–28^.

Several selection-driven hypotheses have been proposed to explain the dichotomy between neurotoxic and hemorrhagic snake venom phenotypes because venom is expected to be locally adapted to prey at a very fine geographic scale^7,29–32^. These hypotheses have centered on potential differences in the digestive efficiency of the two venom types^33^; Type B venoms likely provide more efficient digestion of prey at lower temperatures or when temperature fluctuations are pronounced^8,34^, although evidence for an increase in digestive efficiency in Type B venoms remains controversial (e.g.^33,35,36^). Alternatively, neurotoxic venoms may be particularly advantageous when the chance of prey escaping is high^34^, as would be expected in metabolically active ectothermic prey in warm areas. This is because Type A venoms rapidly subdue prey through neuromuscular paralysis, but do not convey the potential digestive benefits that high SVMP activity does^4^. An additional selection pressure to consider is the types of prey found in regions with alternative venom types, as neurotoxic venom components have very specific targets and are likely effective against select prey items, while Type B venoms may have broad biological effects across more taxonomically diverse prey^4^.

While natural selection is thought to be a major driver of fine-scale geographic differences in venom phenotypes, little attention has been given to the potential role of other evolutionary forces (e.g., genetic drift, gene flow, population structure) in the evolution of the A/B dichotomy in the venom phenotypes of rattlesnakes. Recently, Dowell *et al*.^37^ proposed that Type A and Type B venom polymorphism in rattlesnakes might be maintained by balancing selection, and also that the presence of individuals with Type A + B venom may be the result of introgression between populations dominated by either Type A and Type B phenotypes. Schield *et al*.^38^ found evidence for four distinct geographically structured lineages within *C*. *scutulatus*, and for gene flow between these structured populations. Although sampling has been limited to date, previous studies have demonstrated that all three (i.e., Type A, Type B, and Type A + B) venom phenotypes occur in the three northern *C*. *scutulatus* lineages^19,21,22,39,40^, whereas *C*. *scutulatus salvini* (the most southern lineage) is thought to be exclusively Type A^15,23^. A number of questions remain, including what evolutionary mechanisms may underlie variation in venom phenotypes, and to what degree venom phenotypes are structured across the range of *C*. *scutulatus*.

In this study, we characterize venom phenotypes and genotypes throughout the entire range of *C*. *scutulatus*. We then link these data on venom variation with data on morphological variation, population genetic structure, and environmental variables to identify evolutionary factors that may underlie the polymorphic venom phenotypes in Mojave Rattlesnakes. Our results indicate that balancing selection is unlikely to explain venom variation, but instead, strong directional local selection for different venom phenotypes appears to drive range-wide venom variation. We also find evidence that venom phenotypes are correlated with annual environmental temperature, suggesting that the primary drivers of selection on venom phenotype variation include factors related to environmental temperature, such as snake digestive physiology, prey distribution, or prey physiology.

## Methods

### Ethics statement

Scientific collecting permits in the United States were issued by the State of Arizona Game and Fish Department (SP628489, SP673390, SP673626, SP715023), the California Department of Fish and Wildlife (SC-12985), the New Mexico Department of Game and Fish (3563, 3576) and Texas Parks and Wildlife (SPR-0390-029). In Mexico, collection permits were issued by the Secretaria de Medio Ambiente y Recursos Naturales of the Estados Unidos Mexicanos (SEMARNAT: SGPA/DGVS/03562/15, SGPA/DGVS/01090/17, and FAUT-0015). Interactions with animals were approved by the University of Central Florida’s (UCF) Institutional Animal Care and Use Committee under protocol 13–17 W and followed the American Society of Ichthyologists and Herpetologists ethical guidelines.

### Sample collection and DNA extraction

We collected representatives of all previously identified lineages^38^ of Mojave Rattlesnakes from throughout their distribution. For most samples collected in the field, we obtained venom and tissue. When possible, voucher specimens were created and deposited (Supplemental Table 1 with abbreviations following^41^). Tissues were stored in 95% ethanol or RNAlater and venom was collected and vacuum dried, frozen in liquid nitrogen, and/or stored at −80 °C. We collected a total of 216 individuals: 114 of these had tissue and venom, 34 had only venom, and 68 had only tissue (Supplemental Table 1). Whole genomic DNA was extracted from samples using the Serapure bead extraction protocol of Rohland and Reich^42^ following modifications in Faircloth^43^.

### Reverse-phased High Performance Liquid Chromatography (RP-HPLC) to determine venom type

All venom was vacuum dried or lyophilized prior to use, resuspended in Millipore-filtered water, and centrifuged to remove insoluble debris. We determined protein concentration on a Qubit 3.0 Fluorometer (ThermoFisher Scientific) using the Qubit Protein Assay (ThermoFisher Scientific) following the manufacturer’s protocol. To determine the venom type (A, B, or A + B) of each individual, we used Reverse-phased High Performance Liquid Chromatography (RP-HPLC) based on the protocol in Margres *et al*.^44^. For RP-HPLC, we injected 100 *μ*g of venom onto a Jupiter C18 column (250 × 2 mm; Phenomenex, Torrence, California, USA) using two solvents: 1 = 0.1% trifluoroacetic acid (TFA) in water and 2 = 0.075% TFA in acetonitrile. We used a Beckman System Gold HPLC (Beckman Coulter, Fullerton, California, USA) located in the Florida State University (FSU) Department of Biological Science Analytical Lab. The gradient started with 95% A and 5% B for 5 minutes followed by a 1% per minute linear gradient to 25% B, followed by a 0.25% per minute linear gradient to 55% B, a 2% per minute linear gradient to 75% B, a 14% per minute linear gradient to 5% B and then 5 minutes at the initial conditions all at a 0.2 mL/min flow rate. Run time was 180 minutes for each sample and the effluent was monitored at 220 and 280 nm^45^. Venoms were assayed using RP-HPLC for 148 individuals based on the presence of both subunits of MTX (Type A) and the presence of SVMPs (Type B) based on previous RP-HPLC profiles in *C*. *scutulatus*^24,46^ under these conditions^5^. When venom was available, RP-HPLC was used as the primary means of determining venom type.

### Mojave toxin assay

To confirm venom type from RP-HPLC and to determine venom type when venom was unavailable, we used PCR assays for both subunits of Mojave Toxin (MTXA and MTXB). We amplified two fragments for each subunit using the primers designed by Zancolli *et al*.^47^. These primers were developed to determine MTX presence in *C*. *scutulatus* individuals from Arizona and New Mexico, USA and have also been shown to successfully amplify these fragments from individual *C*. *scutulatus* from Mexico^22^. PCR amplification was conducted on each DNA sample under the following conditions per 10 *μ*L reactions: 3.5 *μ*L PCR water, 1 *μ*L 10X Sigma Buffer (Sigma-Aldrich, St. Louis, MO, USA), 1 *μ*L of 25 mM MgCL_2_ (Sigma-Aldrich), 1.3 *μ*L of 2.5 mM each dNTPs, 0.5 *μ*L of each primer at 10 *μ*M, 0.2 *μ*L of Taq Polymerase (Sigma-Aldrich), and 2 *μ*L DNA. PCR was conducted on PTC200 Thermal Cycler (Bio-Rad, Hercules, CA, USA): 3.5 minutes at 94 °C, 35 cycles of 30 seconds at 94 °C, 1 minute at 57 °C, and 1 minute at 72 °C, and a final extension at 72 °C for 5 minutes. PCR product amplification was evaluated on a 2% agarorose gel using GelRed dye (Biotium, Fremont, CA, USA) to determine if the subunits were present. If one assay was positive, the individual was considered to have MTX and be Type A because all available data suggest that if these loci are present, they are also expressed in the venom^22,47^. We were unable to distinguish Type A and Type A + B individuals using the PCR assay because we could not determine the presence or absence of SVMPs without a venom sample.

### Venom phylogeography of *C*. *scutulatus*

To determine if venom type correlated with phylogenetic lineage, we PCR amplified and sequenced the mitochondrial NADH4 (ND4) gene for any individual in our dataset not already sequenced in Schield *et al*.^38^. As outgroups, we included one sample each from *C*. *viridis*, *C*. *cerberus*, and *C*. *oreganus*, the sister species complex to *C*. *scutulatus*^48^ (Supplemental Table 1). We used the primers ND4 and Leu to sequence the partial ND4 gene as well as the adjacent His, Ser, and Leu tRNA genes^49^. PCR was conducted under the following conditions per 10 *μ*L reaction: 3.8 *μ*L PCR water, 1 *μ*L 10X Sigma Buffer (Sigma-Aldrich), 1 *μ*L of 25 mM MgCL_2_ (Sigma-Aldrich), 0.8 *μ*L of 2.5 mM each dNTPs (Invitrogen, Waltham, Massachusetts, USA), 0.5 *μ*L of each primer at 10 *μ*M, 0.4 *μ*L of Taq Polymerase (Sigma-Aldrich) and 2 *μ*L DNA. PCR was conducted on a PTC200 Thermal Cycler (Bio-Rad): 3.5 minutes at 94 °C, 35 cycles of 30 seconds at 94 °C, 1 minute at 53 °C, and 1 minute at 72 °C, and a final extension at 72 °C for 5 minutes.

Amplicons were purified by adding 0.5 *μ*L FastAP (ThermoFisher Scientific #EF0651), 0.05 *μ*L Exonuclease 1 (ThermoFisher Scientific #EN0581), and 7.45 *μ*L PCR water to each 30 *μ*L reaction and then placed on the thermal cycler for 30 minutes at 37 °C followed by 15 minutes at 85 °C. Sequencing was done in both directions at Eurofins Scientific (St. Charles, Missouri, USA) on an ABI 3730 genetic analyzer (Applied Biosystems, Waltham, MA, USA). Sequences were assembled and edited in Geneious v 10.1.2 (Biomatters Ltd., Auckland, New Zealand). Alignments were created with the MAFFT v 7.22 alignment algorithm^50^ implemented with default parameters in Geneious. We verified alignments by eye and trimmed low quality nucleotides and also checked to ensure there were no frameshift mutations. Our final alignment was 884 nucleotides for 190 ingroup and three outgroup taxa.

We used PartitionFinder 2.1.1^51^ to determine the best-fit model of evolution for the ND4 alignment, partitioned by codon position and between protein-coding and tRNA regions. We used the “greedy” search algorithm, Bayesian Information Criterion (BIC), linked branch lengths, and only tested models available in MrBayes. The starting tree was generated using PhyML v 3.0^52^. These analyses were run in the UCF Advanced Research Computing Center (ARCC) on the Stokes High Performance Computer (SHPC). Best fit models from PartitionFinder2 were HKY + I for the first codon position and the tRNA together, HKY for the second codon position, and HKY + Γ for the third codon position.

To determine the mitochondrial lineage of new samples in reference to Schield *et al*.^38^, we used Bayesian Inference (BI) in MrBayes v 3.2.6^53^. Four independent MCMC runs were conducted for 10^7^ generations and samples taken every 500 generations. The first 25% of each run was discarded as burnin and all runs were checked in Tracer v 1.6^54^ to ensure stationarity was reached and that all ESS values for parameters from the individual and combined runs were ≥200. We combined the runs and generated a 50% majority rule tree.

### Azocasein metalloproteinase assay

To determine if there are differences within and among venom types among populations, we performed an azocasein metalloproteinase assay on 146 samples in triplicate^46^. These assays were performed by incubating 20 *μ*g of venom with 1 mg of azocasein substrate in buffer composed of 50 mM HEPES and 100 mM NaCl at a pH of 8.0 for 30 minutes at 37 °C. We stopped the reaction with 250 *μ*L of 0.5 M trichloroacetic acid, vortexed, and brought it to room temperature. We then centrifuged it at 2000 rpm for 10 minutes. Sample absorbance was read at 342 nm and reported in Δ_342*nm*_/min/mg of venom protein^46^. To determine the statistical significance of differences among samples at an *α* of 0.05, we used a Kruskal-Wallis test with venom type and lineage as factors implemented in R v. 3.4.3^55^. If there was significance globally, we used a Nemenyi post hoc test implemented in the R package PMCMR to determine pairwise significance^56^.

### Kallikrein-like serine protease assay

To test if there are differences within venom types for other toxin classes, we performed a kallikrein-like serine protease assay on 60 samples following the protocol of Mackessy^57^. This assay was conducted by adding 0.8 *μ*g of whole venom to 373 *μ*L of the same buffer as that used for azocasein metalloporoteinase assay described above. Samples were incubated for 3 minutes at 37 °C and then 50 *μ*L of substrate (Bz-ProPheArg-pNA; Bachem, Torrance, CA, USA), the sample was vortexed and placed back at 37 °C for three minutes. The reaction was stopped with 50% acetic acid. Sample absorbance was read at 405 nm and the specific activity was calculated based on a standard curve of p-nitroaniline and reported as nanomoles of product produced per minute per mg of venom^46^. We used a Kruskal-Wallis test with venom type and lineage as factors in R to test for significant differences at an *α* of 0.05. If there was significance globally, we used a Nemenyi post hoc test implemented in the R package PMCMR to determine pairwise significance^56^.

### SDS-PAGE protein gel electrophoresis

To characterize overall venom peptide diversity, we performed SDS-PAGE protein gel electrophoresis on 110 samples following the protocol of Smith and Mackessy^46^. We loaded 20 *μ*g of whole venom into wells of a NuPAGE Novex bis-tris 12% acrylamide mini gel (Life Technologies, Grand Island, NY, USA) and elecrophoresed in MES buffer at 175 volts for 45 minutes. To estimate the molecular weight, we used 7 *μ*L of Mark 12 standard. We stained gels overnight on a gentle shake with 0.1% Coomassie brilliant blue R-250 in 50% and 20% acetic acid (v/v). Gels were destained for approximately two hours in 30% methanol and 7% glacial acetic acid (v/v) in water until bands were clearly visible. Gels were gently shaken overnight at room temperature in 7% acetic acid (v/v) storage solution and imaged the following day using an HP Scanjet 4570c scanner.

### Head morphology analysis

To determine if there are head morphological differences, we measured morphological characters for 57 individuals from Arizona, USA. We followed Margres *et al*.^13^ and measured SVL (snout to vent length), HL (head length), HW (head width), IF (interfang distance), and FL (fang length) for both fangs when not broken and then averaged them. We measured SVL with a tailor’s tape from the tip of the snout to the posterior end of the cloaca to the nearest 1 mm. We used IP54 digital calipers (iGaging, San Clemente, CA, USA) to measure HL, HW, IF, and FL to the nearest 0.01 mm. Head length was measured from the tip of the snout to the articular-quadrate joint, HW was the widest point behind the eye, IF was the distance between the two fang maxillae, and FL was from the top of the maxilla to the tip of the fang while folded. Average FL was determined if neither fang was broken. If one fang was broken, then the unbroken fang was used as the sole measurement; if both fangs were broken, that individual was not included in analyses of FL. All measurements were natural-log-transformed so they met the assumptions of normality and homoscedasticity. To size correct the data, we used the lnSVL value and subtracted each other value from it (ex. lnHL-lnSVL) to generate new columns for HL, HW, IF, and FL that were standardized based on the length of the snake. Using these data, we first compared all individuals based on presence or absence of Mojave Toxin which allowed us to include all samples. We then excluded animals in which we could not distinguish between Type A and Type A + B and compared individuals of the three venom types. All comparisons were done using Kruskal-Wallis tests at an *α* of 0.05. All transformations and analyses of morphological data were done in R v. 3.4.3^55^. If there was significance globally, we used a Nemenyi post hoc test implemented in the R package PMCMR to determine pairwise significance^56^.

### Niche modeling of venom type

To examine whether differences in venom type were correlated with differences in environmental variables, we constructed ecological niche models (ENMs) for the occurrence of Type A and Type B venom across the range of *C*. *scutulatus*. We used geographic localities for 81 type A and 68 type B *C*. *scutulatus*. Individuals that did not have venom but were positive for MTX were excluded from the analysis because we could not differentiate Type A vs Type A + B. ENMs were generated using MAXENT v 3.4.1^58^ implemented in the R package dismo^59^. MAXENT uses occurrence records and a user-provided suite of environmental variables to predict the suitability of habitat and likelihood of occurrence across a landscape^60^. To limit the effect of sampling bias in the construction of EMS, we subsampled the total set of occurrence records to the same resolution as the our environmental data (30 arc seconds). This reduced the dataset to 72 and 54 representative Type A and Type B *C*. *scutulatus* occurrence records, respectively. For environmental data we used the 19 climatic variables collected in the WorldClim dataset v 1.4^61^ as well as elevation, slope, and aspect with a 30 arc second resolution. To avoid biasing model fitting through inclusion of highly correlated inputs^62^, we removed 8 variables (BIO3, BIO5, BIO7, BIO10, BIO11, BIO13, BIO16, and BIO17) with a pair-wise Pearson’s correlation coefficient >0.90. BIO3 and BIO 7 were removed because they are derivatives of other bioclim variables and were correlated with them. BIO5 and BIO10 were removed because they were correlated with elevation. Elevation was kept because it was a variable specific to one of our hypotheses. BIO6 was removed in favor of BIO11 because temperature of the coldest month is less general than temperature of the coldest quarter. BIO13 and BIO16 were removed in favor of BIO12, the was more general. Lastly BIO14 was removed and BIO17 was kept, once again because BIO17 was more general. This left 14 remaining environmental variable characterizing southwestern North America. MAXENT models were run with 20 replicates and average model performance was evaluated by determining Area Under the Curve (AUC) for each model. ENMs were then generated for the Sonoran, Chihuahuan, and Central Mexican Plateau lineages independently following the same methods except only 10 replicates were used due to decreased sample size per lineage. This was done to further examine the correlations between environmental variables and venom types at finer geographic scales.

To test niche equivalency and niche similarity of Type A and Type B individual geographic distributions, we used the pseudoreplicate simulation method of Warren *et al*.^63^ as implemented in the R package phyloclim^64^. Both tests were run with 99 replicates to build a null distribution against which to test values of Schoener’s D and Warren’s I inferred from the full data models. To test niche equivalency, the occurrence points of two populations (e.g., individuals with Type A versus Type B venom) are combined and randomly partitioned into two datasets with sizes equal those of Type A and Type B. ENMs are generated for each dataset (e.g., a Type A model and a Type B model) and their similarity values (D and I) are calculated. This process is repeated to build a null distribution against which we test actual values of D and I inferred from each of the two full data models. The pseudoreplicate simulation method tests the null hypothesis that the two models (Type A and Type B) are not significantly different. In contrast, the test of niche similarity compares the ENMs of one venom type to an ENM of randomly selected subset of background cells (which include both presence and absence locations) of the other species. This is replicated 99 times and is then reversed such that the models for Type A venom are tested against background models for Type B venom and models for Type B venom are tested against background models for Type A venom. Comparison of the true D and I statistics to the pseudoreplicate distributions tests the hypothesis that one venom type’s niche model predicts the occurrences of the other venom type more than expected by chance. In addition, to more directly test for presence and absence differences as relating to specific environmental variables, we used logistic regression on the 14 variables used for ENMs. This approach was used to distinguish variables that may be important for *C*. *scutulatus*’ ecology, but may not be correlated with differences between venom types. Niche equivalency, niche similarity, and logistic regression of the model variables were then estimated for the Sonoran, Chihuahuan, and Central Mexican Plateau lineages independently to determine if the global pattern was the same at finer geographic scales.

### Accession codes

MH883648-MH883754.

## Results

### Venom type in *C*. *scutulatus*

**Figure 2:**
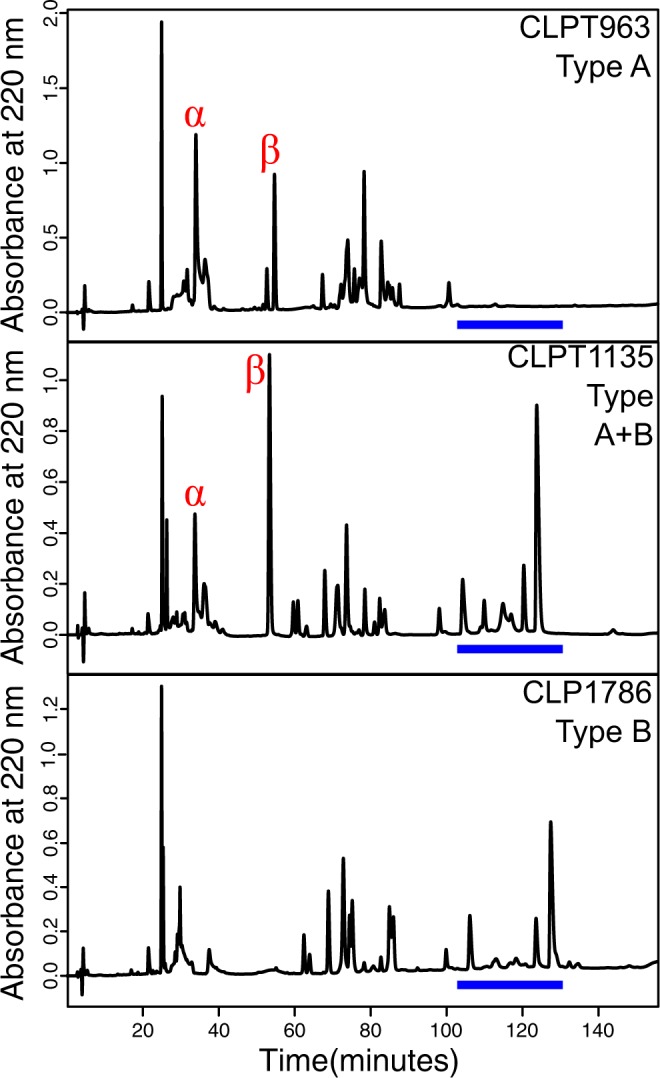
Representative Reverse-phased High Performance Liquid Chromatography (RP-HPLC) profiles of Type A (top), Type A + B (middle), and Type B (bottom) venom of Mojave Rattlesnakes. The acidic (*α*) and basic subunit (*β*) peaks for Mojave toxin are marked and the region where snake venom metalloproteinases elute is marked with a blue bar. Type B individuals lack both subunits of Mojave toxin.

For the 114 samples from which both venom and blood were sampled, the protein-based RP-HPLC venom type assay and the PCR-based MTX assay were in agreement in either detecting or not detecting MTX; no individuals that lacked MTX in the RP-HPLC profile had a positive result in the PCR assays (see representative examples in Fig. 2). All samples that had both subunits of MTX in their venom protein profile were positive for MTX in at least one PCR assay, and all but seven individuals were positive for all four MTX PCR assays. For the 68 samples that only had DNA available, all but five were unanimous for venom type. A total of 12 samples, likely due to DNA quality, were not positive for all four assays: two were positive for three of four assays, seven were positive for two of four, and three were positive for one assay. In total, 144 samples were positive for Mojave Toxin and 72 were negative for Mojave Toxin (Supplemental Table 1). Excluding samples in which Type A and Type A + B could not be distinguished, we had 86 Type A, 51 Type B, and 11 Type A + B (7.43% Type A + B). For the 47 individuals that were positive for the PCR MTX assay, we could not differentiate between Type A and Type A + B. We indicate when these are included in our analyses and when they are excluded.

### Population genetic structure

**Figure 3:**
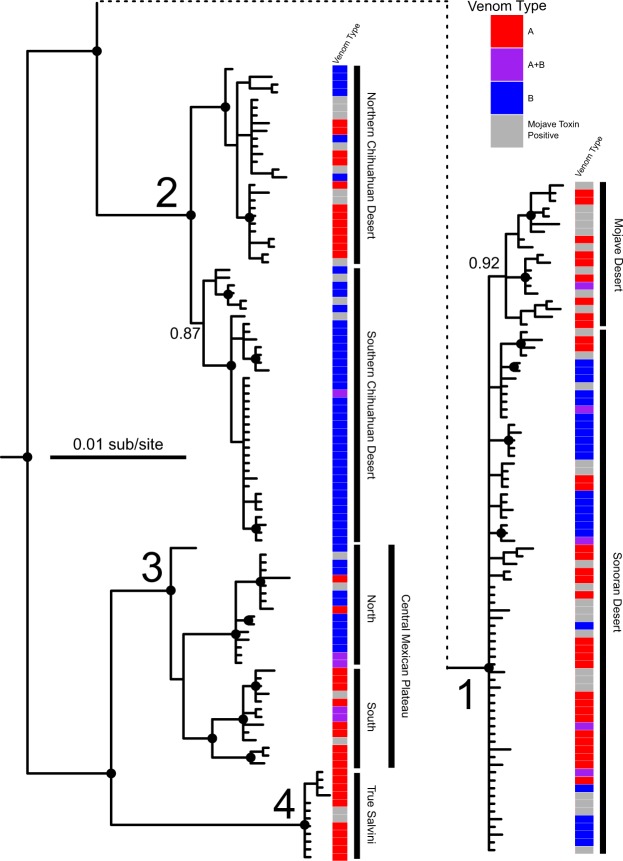
Bayesian Inference phylogeny based on ND4 sequence from 190 *C*. *scutulatus*. The dashed line indicates where the Sonoran and Mojave Desert lineage was moved from and no size adjustments occurred. Venom type as a discrete character is mapped onto the phylogeny. Dots on nodes represent significant posterior probability values of ≥0.95. The four primary clades are numbered as in Fig. 1 and subpopulations are denoted by bars and labeled.

As expected, our mitochondrial ND4 phylogeny of individuals was highly similar to that of Schield *et al*.^38^ in recovering four main clades of haplotypes within *C*. *scutulatus* (Fig. 3). Because of the addition of more samples from the Central Plateau of Mexico, we identified potential substructure in this region (Fig. 3). Based on where each individual was in our phylogeny, we were able to assign each individual to a population and test if there were difference in venom characteristics that corresponded to shared ancestry. For our analyses, we used the three lineages with both Type A and Type B venom to compare populations and venom type. Only the lineage corresponding to *C*. *scutulatus salvini* was monotypic for venom type (Fig. 3).

### Geographic and phylogenetic distribution of venom types

We find that venom types are variable within three of the four major lineages of *C*. *scutulatus*, and that the Type A phenotype is fixed only in the *C*. *scutulatus salvini* lineage (Figs 1 and 3). Within these major lineages, geographically-defined populations tend to be nearly fixed or fixed for a single venom type (i.e., Type A or Type B), and there is very little evidence of populations with highly polymorphic venom composition (Table 1 and Figs 1 and 3). Populations containing individuals with mixed Type A + B venom tend to occur in regions that effectively represent hybrid zones based on Schield *et al*.^38^, as these are regions were estimated to share substantial gene flow. Thus, the populations that exhibit phenotypic polymorphism in venom tend to be those that occur at the interface of populations that exchange gene flow. Otherwise, venom type tends to be fixed in a particular population outside of these zones (Table 1). Intriguingly, even within regions inferred to be panmictic based on nuclear data from Schield *et al*.^38^ (e.g., populations in Arizona; Fig. 1), we observe geographic subpopulations that are differentially fixed for one venom type or the other, and find little evidence for multiple venom types within the same geographically-distinct population (Figs 1 and 3). For example, the northern Chihuahuan Desert (Texas, USA) is fixed for Type A and all but one of the southern Chihuahuan Desert individuals is Type B (Table 1). Where these two lineages come into contact, there is a transition and both venom types occur. Additionally, all but one individual in the Mojave Desert (California and NW Arizona, USA) was Type A (Figs 1 and 3).

**Table 1:** Percentage and number of each venom type for the four lineages as well as the subpopulations within the first three lineages.

	Type A		Type A + B		Type B		MTX+
	%	No.	%	No.	%	No.	
Lineage 1	65.43	53	6.17	5	28.4	23	28
Sonoran	53.45	31	6.9	4	39.66	23	19
Mojave	95.65	22	4.35	1	0	0	9
Lineage 2	20.41	10	2.04	1	77.55	38	13
Northern Chihuahuan	62.5	10	0	0	37.5	6	10
Southern Chihuahuan	0	0	3.03	1	96.97	32	3
Lineage 3	42.86	12	17.86	5	39.29	11	4
North	7.14	1	14.29	2	78.57	11	1
South	78.57	11	21.43	3	0	0	3
Lineage 4 (*salvini*)	100	11	0	0	0	0	2

Additionally, the number of individuals that were positive for the Mojave Toxin (MTX+) assay but could not be distinguished between Type A and Type A + B venom are indicated.

### Venom characterization and activity assays

**Figure 4:**
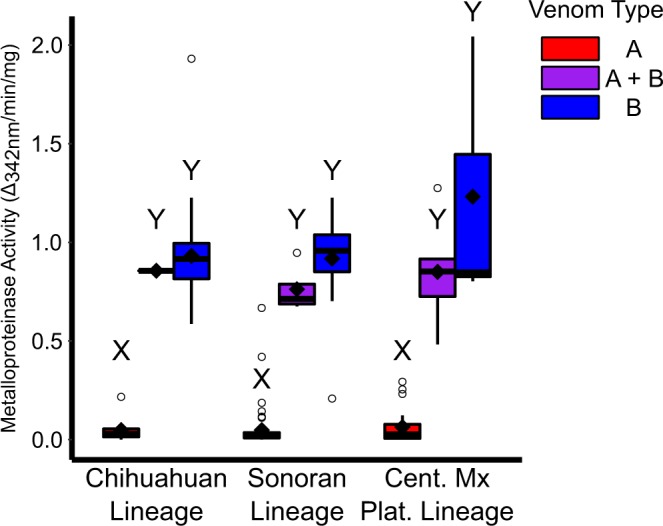
Metalloproteinase activity levels for venom types within each lineage. Type A venom had significantly less metalloproteinase activity regardless of lineage. Type B and A + B were not significantly different from each other in any comparison. Letters above bars (X and Y) indicate significance at an *α* of 0.05.

Metalloproteinase activity was significantly different between venom types (*χ*^2^ = 112.54, df = 2, p < 0.01) with Type A lower than both Type A + B (p < 0.01) and Type B (p < 0.01) and Type A + B and Type B not different (p = 0.11) using the Nemenyi post hoc test. Owing to the higher proportion of Type B individuals in the Chihuahuan Lineage, there was a significant difference among lineages (*χ*^2^ = 24.158, df = 2, p < 0.01) with the Chihuahuan Lineage being higher than the Sonoran (p < 0.01) and Central Mexican Plateau Lineage (p < 0.01) but the latter two not being different from each other (p = 0.94). Metalloproteinase activity was not different across lineages for Type A (*χ*^2^ = 1.33, df = 2, p = 0.515), Type A + B (*χ*^2^ = 0.54, df = 2, p = 0.76), or Type B (*χ*^2^ = 0.79, df = 2, p = 0.67) venom (Fig. 4). Type A venom metalloproteinase activity ranged from 0 to 0.668 Δ_342*nm*_/min/mg (n = 86, Average = 0.052, Median = 0.020), Type A + B from 0.483 to 1.275 Δ_342*nm*_/min/mg (n = 10, Average = 0.816, Median = 0.919), and Type B from 0.208 to 2.043 Δ_342*nm*_/min/mg (n = 50, Average = 0.945, Median = 0.919). Eleven of the Type A individuals had metalloproteinase activity values above 0.1 including six that had higher activity than the lowest Type B individual (CLP1834 = 0.208) but did not have SVMP peaks in their RP-HPLC profiles. These individuals may be functionally more similar to Type A + B individuals.

**Figure 5:**
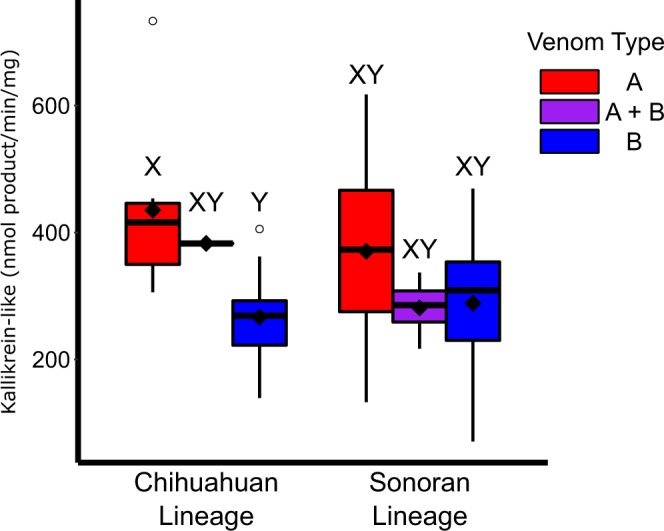
Kallikrein-like serine protease activity between the Chihuahuan and Sonoran lineages. Type B venom had significantly lower activity than Type A venom in the Chihuahuan lineage but there were no differences in the same venom type between the two populations and Type A + B venom was not significantly different from Type A or Type B venom. Letters above bars (X and Y) indicate significance at an *α* of 0.05.

We only had data from the Sonoran and Chihuahuan lineages for Kallikrein-like activity (Fig. 5). Kallikrein-like serine protease activity was highly variable overall and was significantly different among venom types (*χ*^2^ = 8.45, df = 2, p = 0.01) with Type B being significantly lower than Type A (p = 0.01) but no different that Type A + B (p = 0.66) and Type A + B and Type A not being different (p = 0.66). There was no significant difference between the two lineages (*χ*^2^ = 0.24, df = 1, p = 0.62). Within the Chihuahuan lineage, there was significant differences among venom types (*χ*^2^ = 10.05, df = 2, p < 0.01; Fig. 5) with Type B being lower than Type A (p = 0.02) but not significantly different than Type A + B (p = 0.07). Type A and Type A + B were also not different (p = 0.899). Within the Sonoran Lineage, there was no difference among venom types (*χ*^2^ = 3.22, df = 2, p = 0.20; Fig. 5). Overall, Type A values ranged from 132.77 to 733.20 nmol product/min/mg (n = 28, Average = 386.58, Median = 380.10), Type A + B from 216.87 to 382.77 nmol product/min/mg (n = 5, Average = 301.54, Median = 298.30), and Type B from 70.69 to 469.24 nmol product/min/mg (n = 27, Average = 277.231, Median = 285.86).

SDS-PAGE confirmed venom type for all samples. The basic subunit of MTX was clear at 14 kD in Type A and Type A + B individuals and absent in Type B individuals (Figs S1 and S2). Additionally, SVMPs were clearly identifiable in Type B and Type A + B individuals at 55 kD and ~22 kD and absent in Type A individuals. Myotoxins at 6 kD were found in 56 of 110 individuals tested and were generally linked with Type A individuals in the Sonoran lineage and Type B individuals in the Chihuahuan lineage. SDS-PAGE illustrated additional diversity in C-type lectins and non MTX Phospholipase A_2_s as well as the uniformity in snake venom serine proteases and cysteine rich secretory proteins (Figs S1 and S2).

### Morphological analysis

**Figure 6:**
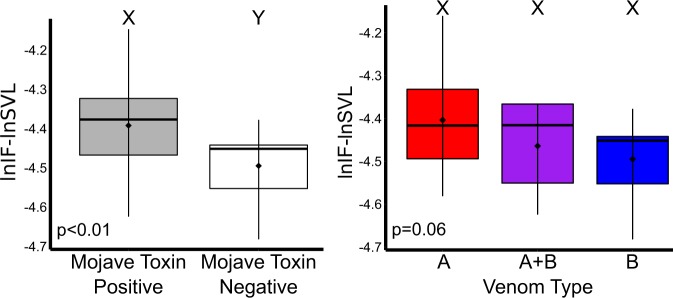
Comparison of Interfang Distance (IF) based on presence and absence of Mojave Toxin (left) and by venom type (right) in the Sonoran lineage of Mojave Rattlesnakes. Snout-vent Length (SVL) was used to control for different sizes among animals. Smaller values on the y-axis (more negative) are smaller measurements. Mojave Toxin positive individuals had significantly wider distances between their fangs compared to Mojave Toxin negative individuals. After excluding animals in which Type A and Type A + B could not be distinguished because they were salvaged, there was marginal significance of interfang distance based on venom type with a trend of Type A individuals having a wider distance between fangs. Letters above bars (X and Y) indicate significance at an *α* of 0.05.

All 57 individuals used in this analysis were from the Sonoran lineage. Thus, they are all genetically similar and provide the best comparison of potential morphological differences associated with the presence of MTX and venom type. We removed eight individuals that were salvaged when comparing venom type because we could not determine if they were Type A or Type A + B. We did not find significant differences between head width based on the presence of MTX (*χ*^2^ = 0.72, df = 1, p = 0.40) or based on venom type (*χ*^2^ = 0.09, df = 2, p = 0.96). Head length was also not significant based on MTX presence (*χ*^2^ = 1.52, df = 1, p = 0.22) or venom type (*χ*^2^ = 1.53, df = 2, p = 0.46). We did find a significant difference in interfang distance based on the presence of MTX (*χ*^2^ = 7.12, df = 1, p < 0.01) but only marginal significance based on venom type (*χ*^2^ = 5.59, df = 2, p = 0.06; Fig. 6). Mojave Toxin positive individuals had a larger distance between the fangs than Mojave Toxin negative individuals. Additionally, there was a trend for MTX positive individuals to have longer fangs than MTX negative individuals (*χ*^2^ = 2.02, df = 1, p = 0.15) and for Type A individuals to have longer fangs than Type A + B and Type B individuals (*χ*^2^ = 3.97, df = 2, p = 0.14).

### ENMs between venom type

**Figure 7:**
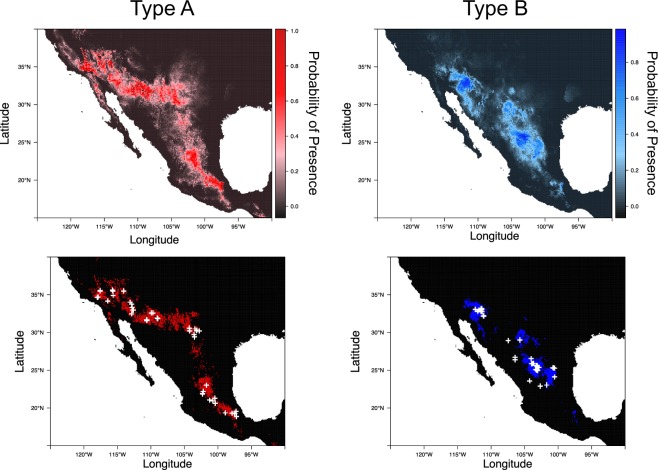
Ecological Niche Models generated in MAXENT^58^ using 72 Type A (left) and 54 Type B (right) Mojave Rattlesnakes scaled by probability of presence (pp). Lower maps display model distributions where each venom type is expected to occur based on a threshold point where model sensitivity and specificity are highest (pp > 0.48 for A’s, pp > 0.24 for B’s).

**Figure 8:**
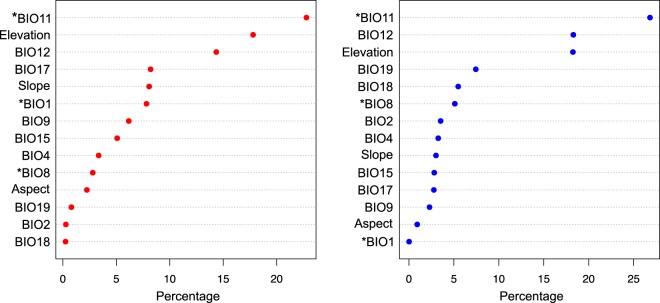
Model response to variables included in the Ecological Niche Models from MAXENT^58^ for Type A (left) and Type B (right) venoms. Asterisks (*) indicate variables that were significantly different between the two models. BIO1 = Annual Mean Temperature, BIO2 = Mean Diurnal Range (Mean of monthly (max temp − min temp)), BIO4 = Temperature Seasonality (standard deviation *100), BIO8 = Mean Temperature of Wettest Quarter, BIO9 = Mean Temperature of Driest Quarter, BIO11 = Mean Temperature of Coldest Quarter, BIO12 = Annual Precipitation, BIO15 = Precipitation Seasonality (Coefficient of Variation), BIO17 = Precipitation of Driest Quarter, BIO18 = Precipitation of Warmest Quarter, BIO19 = Precipitation of Coldest Quarter.

We found significant differences between the variables, particularly in regard to temperature for the ecological niche models (ENM) created for Type A and Type B individuals (Table 2 and Fig. 7). The ENM for Type A and Type B were not equivalent to each other (Fig. S3) but they were more similar than would be expected by chance (Fig. S7). Area under the curve (AUC) from comparison of the model and the Type B (AUC = 0.987) *C*. *scutulatus* was comparable to AUC’s from Type A test data (AUC = 0.907), indicating similarity in predictive power. The WorldClim bioclimatic variable BIO11 (Minimum temperature of the coldest quarter) explained the most variation for both the Type A and Type B models and was significantly different between the two models (Fig. 8 and Table 2). The other variables that were significantly different were BIO1 (annual mean temperature) and BIO8 (mean temperature of wettest quarter); the contribution of these variables differed between models (Fig. 8 and Table 2).

**Table 2:** Logistic regression comparison of the 14 variables used in the Type A and Type B models for *C*. *scutulatus*.

Independent Variable	All Samples			Sonoran			Chihuahuan			Cent. MX Plat.		
	Df	F-value	P-value	Df	F-value	P-value	Df	F-value	P-value	Df	F-value	P-value
BIO1	124	−4.988	<**0**.**001**	56	−3.691	<**0**.**001**	39	−2.622	**0**.**008**	19	1.852	0.064
BIO2	124	−1.583	0.114	56	−1.637	0.102	39	−0.435	0.663	19	0.637	0.524
BIO4	124	1.383	0.167	56	−1.748	0.081	39	1.264	0.206	19	−2.257	**0**.**024**
BIO8	124	−3.411	<**0**.**001**	56	−2.480	**0**.**013**	39	−0.285	0.775	19	0.416	0.677
BIO9	124	−0.780	0.435	56	−3.212	**0**.**001**	39	−2.766	**0**.**006**	19	1.912	0.056
BIO11	124	−5.045	<**0**.**001**	56	−3.696	<**0**.**001**	39	−3.272	**0**.**001**	19	2.107	**0**.**035**
BIO12	124	0.654	0.513	56	−2.226	**0**.**026**	39	1.395	0.163	19	1.782	0.075
BIO15	124	−0.515	0.607	56	3.276	**0**.**001**	39	−2.604	**0**.**009**	19	2.115	**0**.**034**
BIO17	124	−0.882	0.378	56	−3.132	**0**.**002**	39	3.368	<**0**.**001**	19	−1.282	0.200
BIO18	124	−1.296	0.195	56	−0.879	0.379	39	0.383	0.701	19	0.781	0.435
BIO19	124	1.167	0.243	56	−2.460	**0**.**014**	39	1.064	0.287	19	−2.190	**0**.**029**
Aspect	124	1.369	0.171	56	−0.995	0.320	39	2.444	**0**.**015**	19	−0.250	0.802
Elevation	124	0.575	0.565	56	3.308	<**0**.**001**	39	−1.039	0.299	19	−1.309	0.190
Slope	124	0.115	0.909	56	2.172	**0**.**030**	39	0.183	0.855	19	−0.60	0.549

We used a full model and then compared the three lineages for which Type A and Type B individuals were identified. Bolded P-values were significantly different between the two models in a comparison. BIO1–BIO9 correspond to measures related to temperature and BIO12-BIO19 correspond to measures related to precipitation.

Lineage-specific ENMs varied in both the differentiation of models and in the variables that differed between venom types (Figs S3–S16, Table 2). Lineage-specific tests of correlation between environmental variables showed high variation in the variables found to be significantly different between venom types and the direction of their effects (Table 2). However, BIO11 was significant in all models and BIO1 was found to be highly significant across the full model, the Sonoran lineage model, and the Chihuahuan lineage and marginally significant for the Central Mexican Plateau lineage (Table 2).

## Discussion

The venom phenotype dichotomy exhibited in Mojave Rattlesnakes was first described in the 1930’s based on different symptoms of snakebite^65^. Subsequent studies of the distribution of venom types led to the conclusion that *C*. *scutulatus* has neurotoxic venom through the majority of their range^15,21,27,37^. Our data indicate that this century-long generalization is not accurate; both Type A and Type B phenotypes occur throughout the distribution of this species, and the geographic pattern of venom composition is much more complex than previously thought (Fig. 1). In this study, we provide the first range-wide analyses of venom composition variation in *C*. *scutulatus*, and our integration of venom characterization with new data on population structure have synergistically increased our ability to understand the patterns of evolution underlying and perpetuating the venom dichotomy in this species.

Previous studies examining the distribution of venom types in *C*. *scutulatus* lacked any population genetic context with which to interpret range-wide variation in venom. Schield *et al*.^38^ identified four major lineages within *C*. *scutulatus*, each of which shares gene flow with adjacent lineages, and within which there is inferred near-panmixia. In this study, we have demonstrated that all but one of the major phylogeographic lineages identified by Schield *et al*.^38^ possess Type A, Type A + B, and Type B individuals (Table 1 and Figs 1 and 3); the *C*. *scutulatus salvini* lineage appears fixed for Type A venom based on 13 individuals sampled. Combining our venom characterizations with population genetic patterns in *C*. *scutulatus* highlights two key features of venom variation within this system. The first is that, in the three lineages that possess each of the venom types, individuals with Type A + B venom tend to occur in regions where Type A and Type B venom types come into contact (Fig. 1). In one case, this corresponds to an area of introgression where the Chihuahuan and Central Mexican Plateau lineages contact one another and exchange genes^38^ (Fig. 1). This region contains a high concentration of Type A + B individuals. Collectively, these patterns suggest that Type A + B venom may represent a product of introgression between venom types rather than local selection for both types. The second key feature is that populations identified in Schield *et al*.^38^ appear to contain geographically segregated subpopulations that are differentially fixed for either Type A or Type B venom (Table 1 and Figs 1 and 3).

Since the discovery of the *C*. *scutulatus* venom system, different mechanisms have been speculated to drive the observed geographic variation in venom^15–18^. One mechanism that has been proposed to explain this apparently maintained Type A and Type B venom dichotomy is balancing selection (e.g., Dowell^37^). Our results provide convincing evidence against balancing selection in favor of differential local selection driving venom variation in *C*. *scutulatus*. While balancing selection should lead to frequent occurrences of observing both Type A and Type B individuals in a single population^66–68^, our data highlight a very different pattern in which geographically segregated subpopulations are largely fixed for a single venom type (i.e., Type A or Type B populations; Table 1). This pattern is instead most consistent with strong directional local selection that differs across populations by favoring one venom type over the other (Fig. 1), and that differential local selection may occur at a very fine geographic scale. We also find evidence that directional selection likely counteracts introgression resulting in mixed venom phenotypes, despite substantial gene flow within and between major *C*. *scutulatus* lineages. This is supported by the apparent rarity of the mixed venom phenotype across the broad range of *C*. *scutulatus* (i.e., only 11 of 148 individuals with Type A + B venom based on proteomic data). This pattern suggests that selection may be quite efficient in maintaining not only one venom type over the other, but also efficient in limiting introgression of genomic regions underlying the mixed venom phenotype.

A primary goal of this study was to identify biotic and abiotic factors that may predict the geographic variation in venom composition. We found evidence for correspondence between the presence or absence of Mojave Toxin and other trophic factors (e.g., inter-fang distance) within the Sonoran lineage (Fig. 6), suggesting possible selection on the integrated components of trophic function (e.g., Margres *et al*.^13^) to accommodate different prey dimensions and possibly different species. This suggests co-evolution, and potentially local and differential selection on trophic function that incorporates both venom and fang morphology. However, because we were only able to test this in one lineage, additional comparisons between these features in other *C*. *scutulatus* lineages, or other rattlesnake lineages, would be useful for testing our hypothesis and determining if there are broad patterns of co-evolution between venom and other trophic phenotypes or if other evolutionary mechanisms, such as drift, are acting on head morphology. If so, these data would further suggest that venom phenotype, along with other trophic adaptations, may be driven by local selection for different prey types or feeding behaviors.

We also found evidence for correlations between environmental variables, especially those related to temperature, (Table 2 Fig. 8) and venom phenotype. A correspondence between venom phenotype and climate is also recapitulated by the surprising evidence for distinct ecological niches for Type A and Type B phenotypes (Fig. 7). Moreover, the spatial distinction between Type A and Type B animals remained clear for Sonoran and Chihuahuan lineages. Though the specific variables of greatest contribution and significant difference between venom types varied across lineage specific models, this likely reflects the broad environmental heterogeneity across the range of *C*. *scutulatus*. The consistent recovery of temperature related variables, especially annual mean temperature and minimum temperature of the coldest quarter, as differing between venom types despite the broader environmental differences underscores its apparent relevance to the venom phenotype dichotomy (Table 2). While the precise link between environmental temperature parameters and venom composition is not clear, these environmental variables may be predictive of other important factors driving selection for different venom phenotypes. For example, annual temperature in different regions throughout the range of *C*. *scutulatus* may be a strong predictor of prey species distributions, behaviors, and physiological activities. These features of local prey populations may in turn impose substantial selection pressures on the venom of *C*. *scutulatus* populations^29,30,47^. A second but non-exclusive possibility is that venom composition and temperature are linked because Type B venom may be beneficial in cooler areas due to its potential digestive benefits^8,33^, including more rapid digestion by these ectothermic predators of prey in cooler conditions.

Perhaps one of the most surprising findings in our study is the apparent rarity of Type A + B venom in *C*. *scutulatus*, despite evidence for the otherwise strong selection for different venom types in different geographic regions. This rarity is intriguing because both major components in this dichotomy each have positive benefits^4,6,69^, and the presence of both could potentially have additive benefits for securing prey. The finding that Type A + B individuals are rare suggests, instead, that there are negative fitness consequences when both neurotoxic and hemorrhagic components are present and expressed within the venom of same individual. This conclusion is strengthened by consideration of the broad trend in venom phenotypes within the entire clade of rattlesnakes^4,8^. Across *Sistrurus* and *Crotalus* species, venom phenotype is either type I or type II (analogous to Type B and Type A in *C*. *scutulatus* venoms, respectively), with essentially no intermediate examples^8^. Why might this be? Neonate rattlesnakes often show an ontogenetic shift in composition from a type II venom to a type I venom as adults^34^, and type II venoms persisting into adulthood (*C*. *oreganus concolor*, *C*. *scutulatus*, *C*. *tigris* etc.) have been suggested to result from paedomorphosis^70^. This provides an intriguing new perspective on venom evolution that is often not considered. Specifically, while patterns of selection with respect to venom evolution are often viewed in the context of positive selection, our results, in light of the above trends, indicate that negative selection against “intermediate” or “mixed” venoms also play an important role in the evolution of venom composition.

## Conclusion

Our characterization of *C*. *scutulatus* venom phenotypes throughout its range has provided among the most complete appraisals of venom variation in this species to date, and provides new interesting questions for the study of venom variation and evolution overall. We found that the geographic pattern of the three venom phenotypes in *C*. *scutulatus* is much more complex than previously hypothesized, and were able to rule out the fixation of venom phenotypes in major *C*. *scutulatus* lineages. Instead, fixation of venom phenotypes occurs at a much more fine geographic scale. The integration of venom phenotype data with recent data on population structure and gene flow provides evidence that venom variation in *C*. *scutulatus* is not likely the product of balancing selection, but instead likely representative of strong directional local selection acting on distinct venom loci (PLA_2_ and SVMP gene clusters) in geographically highly differentiated ways. We also find evidence that the mixture of venom phenotypes is likely the result of introgression between populations fixed for Type A and Type B venom, and that this admixture is rare. Links between venom phenotypes, fang morphology, and climatic variables represent intriguing areas for further study to determine the broad relevance of these features in shaping venom evolution.

## Electronic supplementary material

Supplemental Figures 1-16

Supplemental Table 1

## Data Availability

New ND4 sequences generated in this study were deposited in GenBank under accession numbers MH883648-MH883754. Specimen vouchers were deposited in the appropriate museums based on permit requirements. All specimen data including morphology and assay data are listed in Supplemental Table 1.
