# Supplementary material for: Evidence for divergent patterns of local selection driving venom variation in Mojave Rattlesnakes (Crotalus scutulatus)
Source: Sci Rep. 2018 Dec 4;8:17622. doi: 10.1038/s41598-018-35810-9 (PMC6279745; doi:10.1038/s41598-018-35810-9)
Supplement: Supplementary file 1 — Supplemental Figures 1-16 [file 41598_2018_35810_MOESM1_ESM.pdf]

Evidence for divergent patterns of local selection driving venom variation in Mojave Rattlesnakes (*Crotalus scutulatus*)

Jason L. Strickland[1,+], Cara F. Smith[2], Andrew J. Mason[3], Drew R. Schield[4], Miguel Borja[5], Gamaliel Castañeda-Gaytán[5], Carol L. Spencer[6], Lydia L. Smith[6], Ann Trápaga[6], Nassima M. Bouzid[7], Gustavo Campillo-García[8], Oscar A. Flores-Villela[8], Daniel Antonio-Rangel[9], Stephen P. Mackessy[2], Todd A. Castoe[4], Darin R. Rokyta[10], and Christopher L. Parkinson[1,+,*]

[1]Department of Biology, University of Central Florida, 4110 Libra Drive, Orlando, FL 32816, USA; Jason.Strickland@knights.ucf.edu

[2]School of Biological Sciences, 501 20th Street, University of Northern Colorado, Greeley, CO 80639 USA; smit4524@bears.unco.edu, stephen.mackessy@unco.edu

[3]Department of Biological Sciences, Clemson University, 190 Collings St., Clemson, SC 29634, USA; ajmason@clemson.edu

[4]Department of Biology, 501 S. Nedderman Drive, University of Texas at Arlington, Arlington, TX 76010 USA; dschield@uta.edu, Todd.Castoe@uta.edu

[5]Facultad de Ciencias Biológicas, Universidad Juárez del Estado de Durango, Av. Universidad s/n. Fracc. Filadelfia, C.P. 35070, Gómez Palacio, Dgo., México; alessandro_53@hotmail.com, gamaliel.cg@gmail.com

[6]Museum of Vertebrate Zoology, 3101 Valley Life Sciences Building, University of California, Berkeley, CA 94720 USA; atrox@berkeley.edu, lydsmith@berkeley.edu, atrapaga@berkeley.edu

[7]Department of Biology and Burke Museum of Natural History and Culture, University of Washington, Box 351800, Seattle, WA 98195 USA; bouzidnm@uw.edu

[8]Museo de Zoología, Department of Evolutionary Biology, Faculta de Ciencias, Universidad Nacional Autónoma de México, External Circuit of Ciudad Universitaria, México City, México; gcampillog@gmail.com, ofvq@unam.mx

[9]Instituto Politécnico Nacional, Escuela Nacional de Ciencias Biológicas, Laboratorio de Cordados Terrestres, Colección Herpetológica, Del. Miguel Hidalgo, México City, México; biologo666@hotmail.com

[10]Department of Biological Science, Florida State University, Tallahassee, Florida 32306, USA; drokyta@bio.fsu.edu

[+]Current address: Department of Biological Sciences & Department of Forestry and Environmental Conservation, Clemson University, 190 Collings St., Clemson, SC 29634, USA

[*]Correspondence: viper@clemson.edu; Tel.: +1-864-656-3058

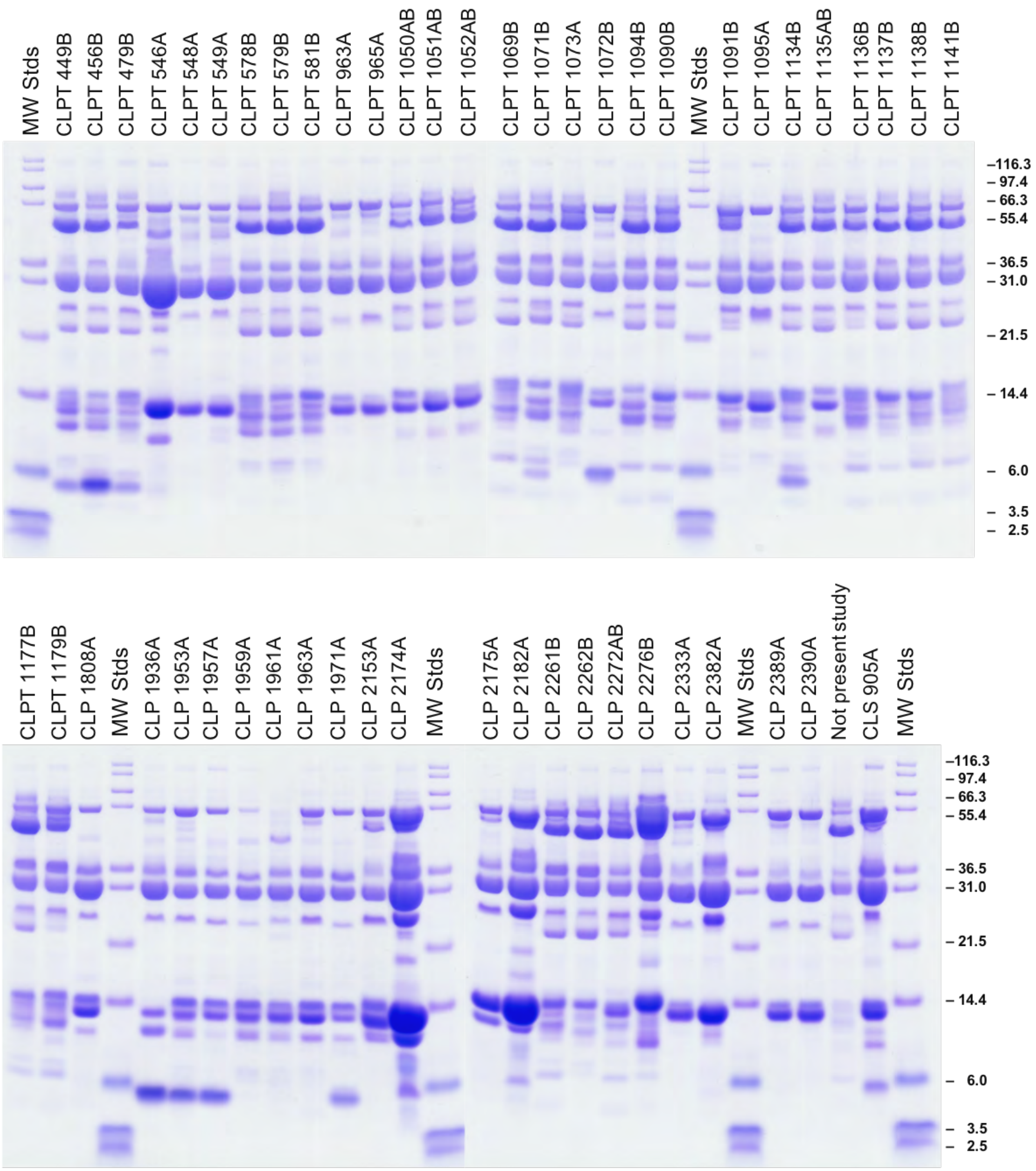

Figure S1. SDS-PAGE gel images for 51 of 110 samples. Venom type of each individual is added to the end of the sample name. When present, the basic subunit of Mojave toxin is at approximately 14kD. See Figure S2 for remaining sample images. Individuals of other species not used in this study are labeled as "Not present study".

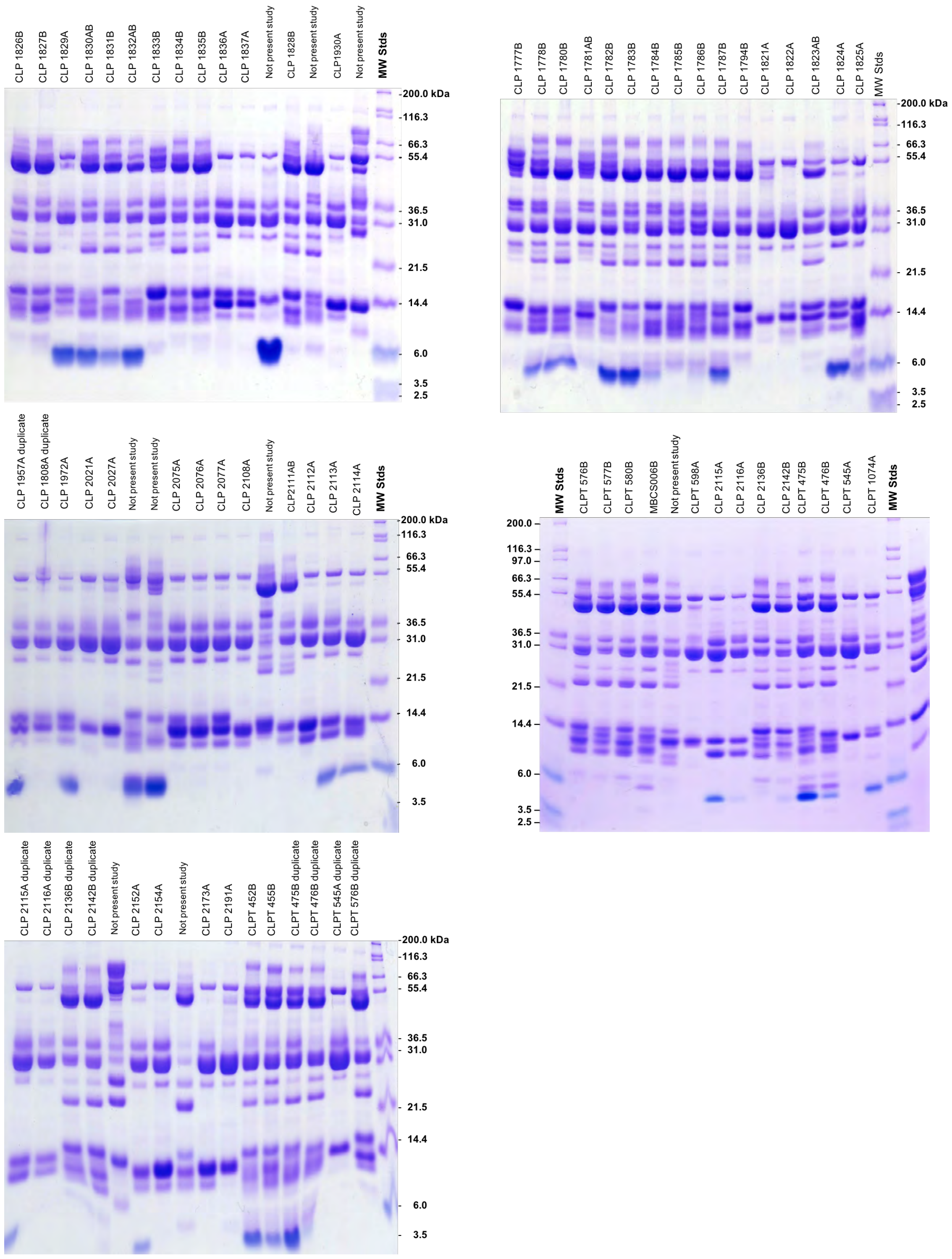

Figure S2. SDS-PAGE gel images for 59 of 110 samples. Venom type of each individual is added to the end of the sample name. When present, the basic subunit of Mojave toxin is at approximately 14kD. See Figure S1 for remaining sample images. Individuals of other species not used in this study are labeled as "Not present study".

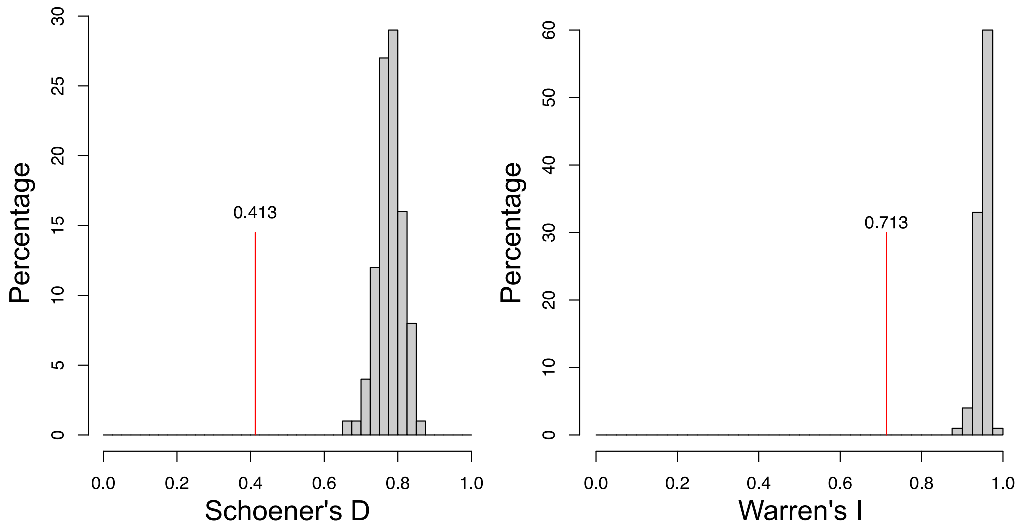

Figure S3. Null distributions generated to test niche equivalency from MAXENT[58] using 99 permutations for all samples. Results for both Schoener's D and Warren's I reject the null hypotheses that the models for Type A and Type B are identical.

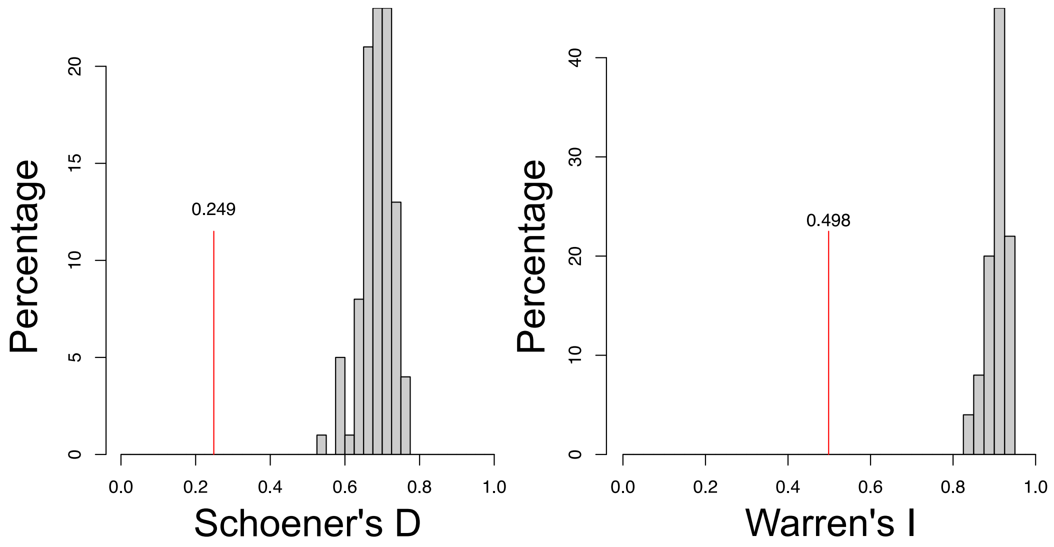

Figure S4. Null distributions generated to test niche equivalency from MAXENT[58] using 99 permutations for individuals from the Sonoran lineage. Results for both Schoener's D and Warren's I reject the null hypotheses that the models for Type A and Type B are identical.

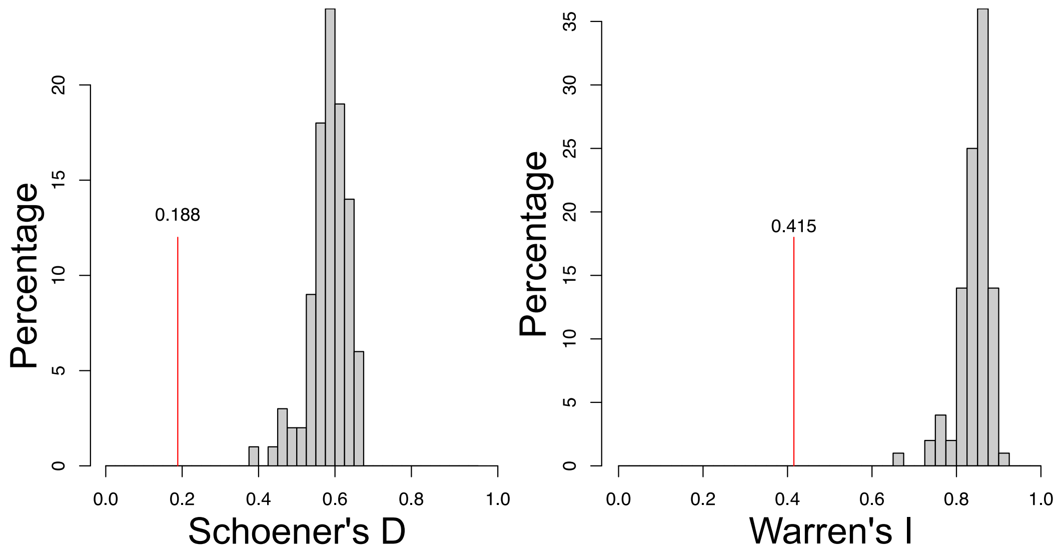

Figure S5. Null distributions generated to test niche equivalency from MAXENT[58] using 99 permutations for individuals from the Chihuahuan lineage. Results for both Schoener's D and Warren's I reject the null hypotheses that the models for Type A and Type B are identical.

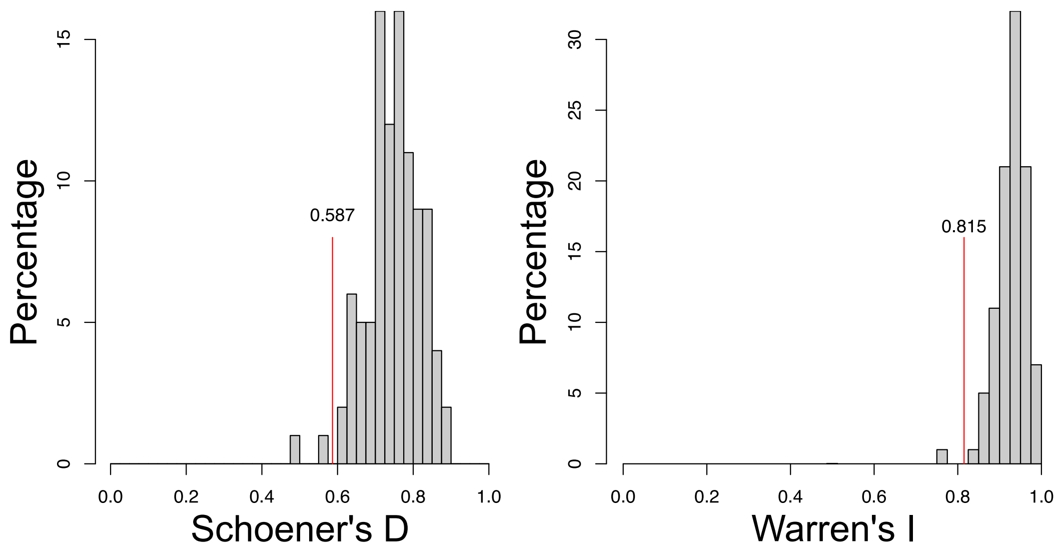

Figure S6. Null distributions generated to test niche equivalency from MAXENT[58] using 99 permutations for individuals from the Central Mexican Plateau lineage. Results for both Schoener's D and Warren's I reject the null hypotheses that the models for Type A and Type B are identical.

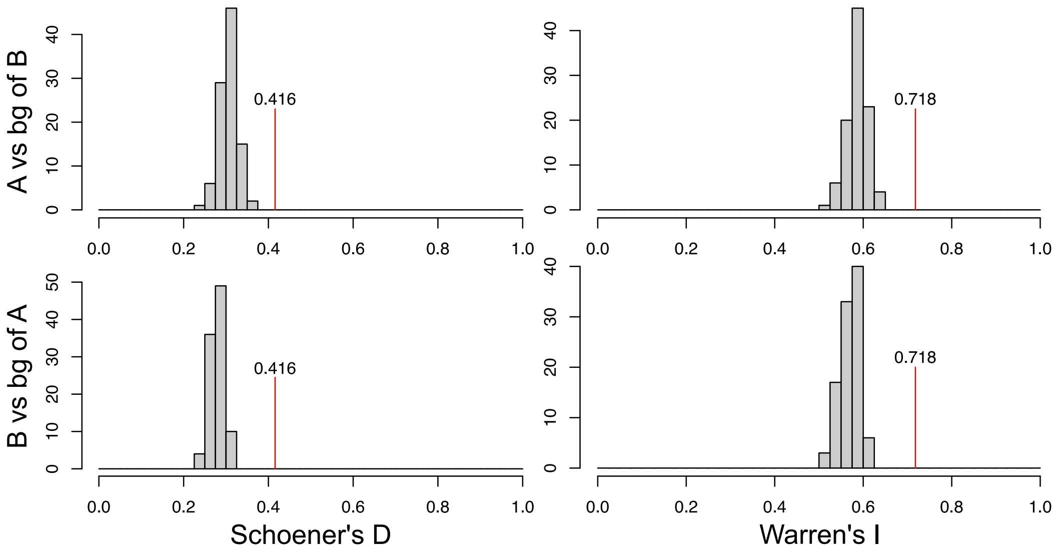

Figure S7. Null distributions generated using 99 permutations from MAXENT[58] of all Type A and Type B individuals to test niche similarity. The top row compares Type A individuals using the model background (bg) of Type B and the bottom row compares Type B individuals using the model background of Type A. Results for both Schoener's D and Warren's I reject the null hypothesis that the two models do not predict the occurrence of each other better or worse than would be expected by chance. Because the D and I values fall in the right tail of the distribution, the niches are more similar than would be expected by chance.

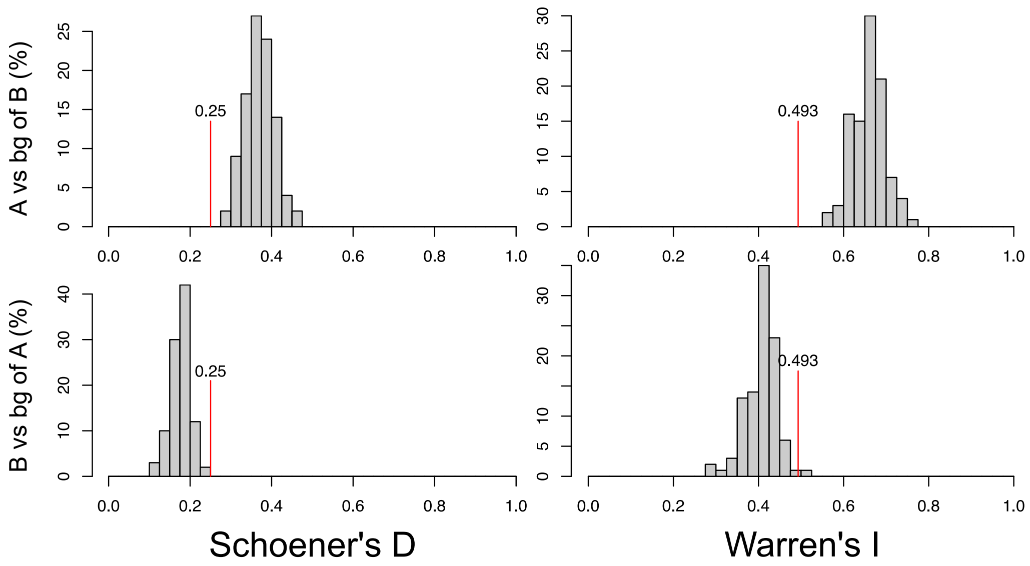

Figure S8. Null distributions generated using 99 permutations from MAXENT[58] of Type A and Type B individuals from the Sonoran lineage to test niche similarity. The top row compares Type A individuals using the model background (bg) of Type B and the bottom row compares Type B individuals using the model background of Type A. Results for both Schoener's D and Warren's I reject the null hypothesis that the two models do not predict the occurrence of each other better or worse than would be expected by chance. The background of B predicts the occurrence of A worse than chance and the background of A predicts the occurrence of B better than chance.

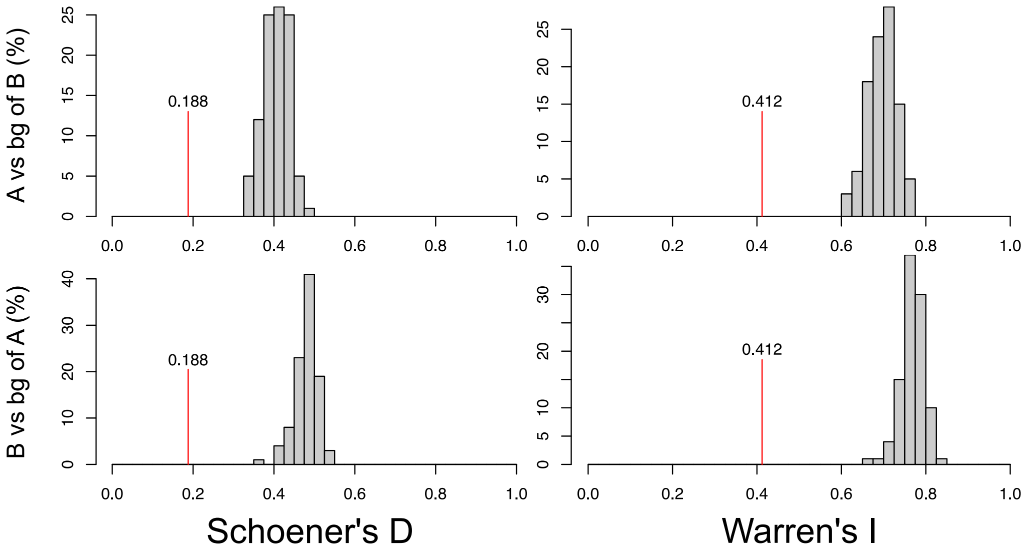

Figure S9. Null distributions generated using 99 permutations from MAXENT[58] of Type A and Type B individuals from the Chihuahuan lineage to test niche similarity. The top row compares Type A individuals using the model background (bg) of Type B and the bottom row compares Type B individuals using the model background of Type A. Results for both Schoener's D and Warren's I reject the null hypothesis that the two models do not predict the occurrence of each other better or worse than would be expected by chance. Because the D and I values fall in the left tail of the distribution, the niches are less similar than would be expected by chance.

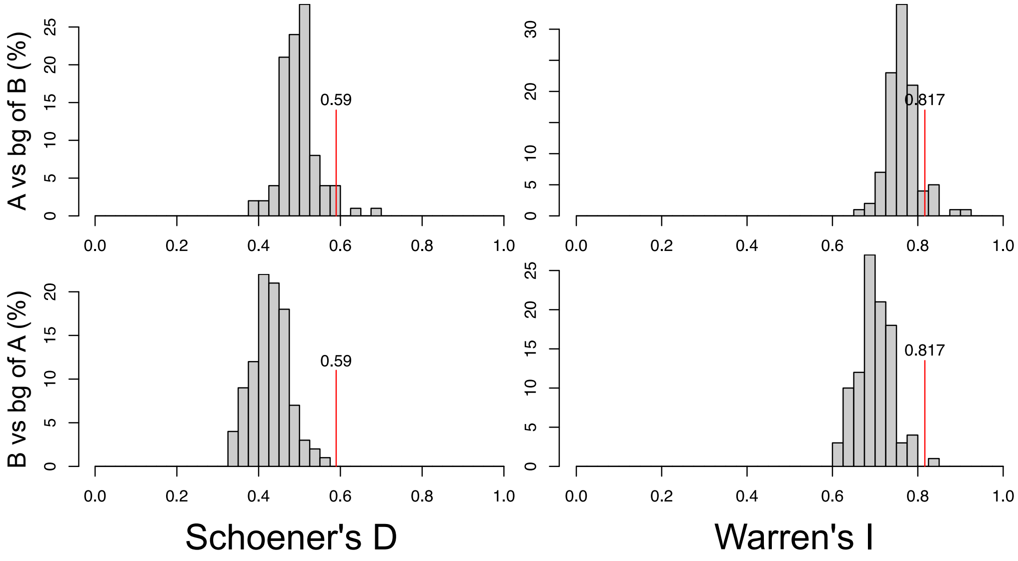

Figure S10. Null distributions generated using 99 permutations from MAXENT[58] of Type A and Type B individuals from the Central Mexican Plateau lineage to test niche similarity. The top row compares Type A individuals using the model background (bg) of Type B and the bottom row compares Type B individuals using the model background of Type A. Results for both Schoener's D and Warren's I fail to reject the null hypothesis that the A vs bg of B model (top) does not predict the occurrence of each other better or worse than would be expected by chance. However, the B vs bg of A model did reject the null and because the D and I values fall in the right tail of the distribution, the niches are more similar than would be expected by chance.

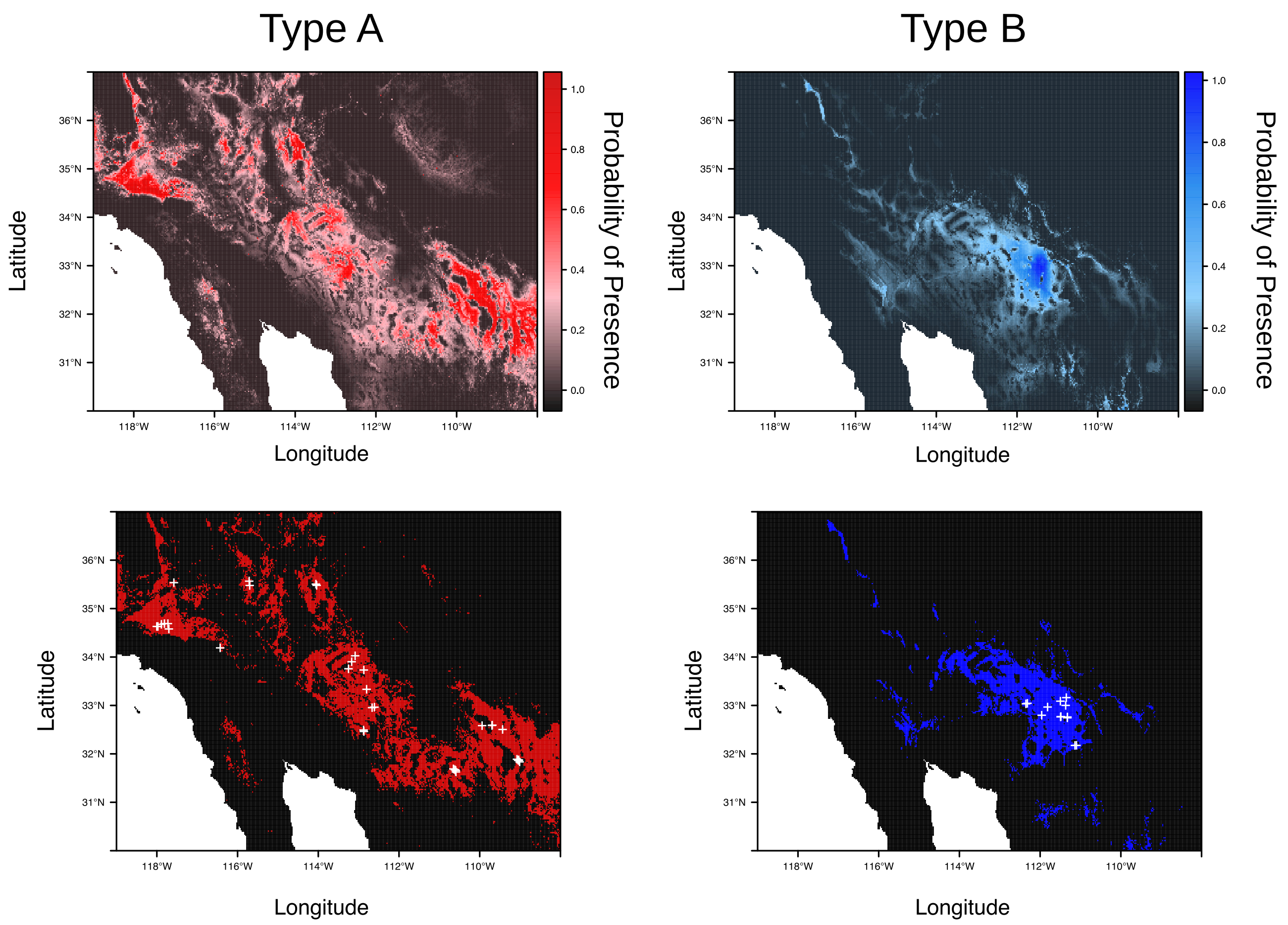

Figure S11. Ecological Niche Models generated in MAXENT[58] using 42 Type A (left) and 16 Type B (right) Mojave Rattlesnakes from the Sonoran lineage scaled by probability of presence (pp). Lower maps display model distributions where each venom type is expected to occur based on a threshold point where model sensitivity and specificity are highest (pp > 0.21 for A's, pp > 0.13 for B's).

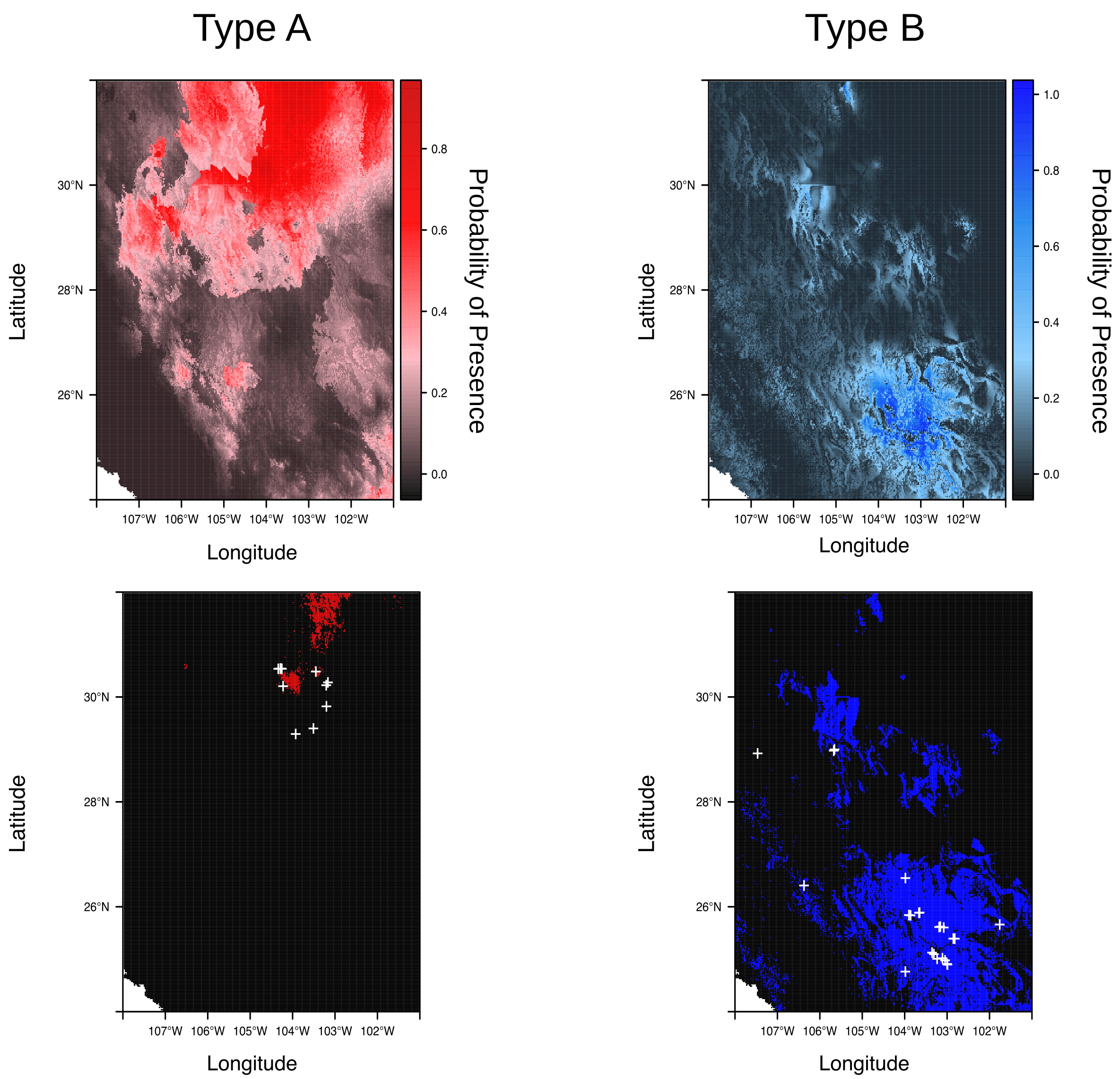

Figure S12. Ecological Niche Models generated in MAXENT[58] using 12 Type A (left) and 29 Type B (right) Mojave Rattlesnakes from the Chihuahuan lineage scaled by probability of presence (pp). Lower maps display model distributions where each venom type is expected to occur based on a threshold point where model sensitivity and specificity are highest (pp > 0.77 for A's, pp > 0.12 for B's).

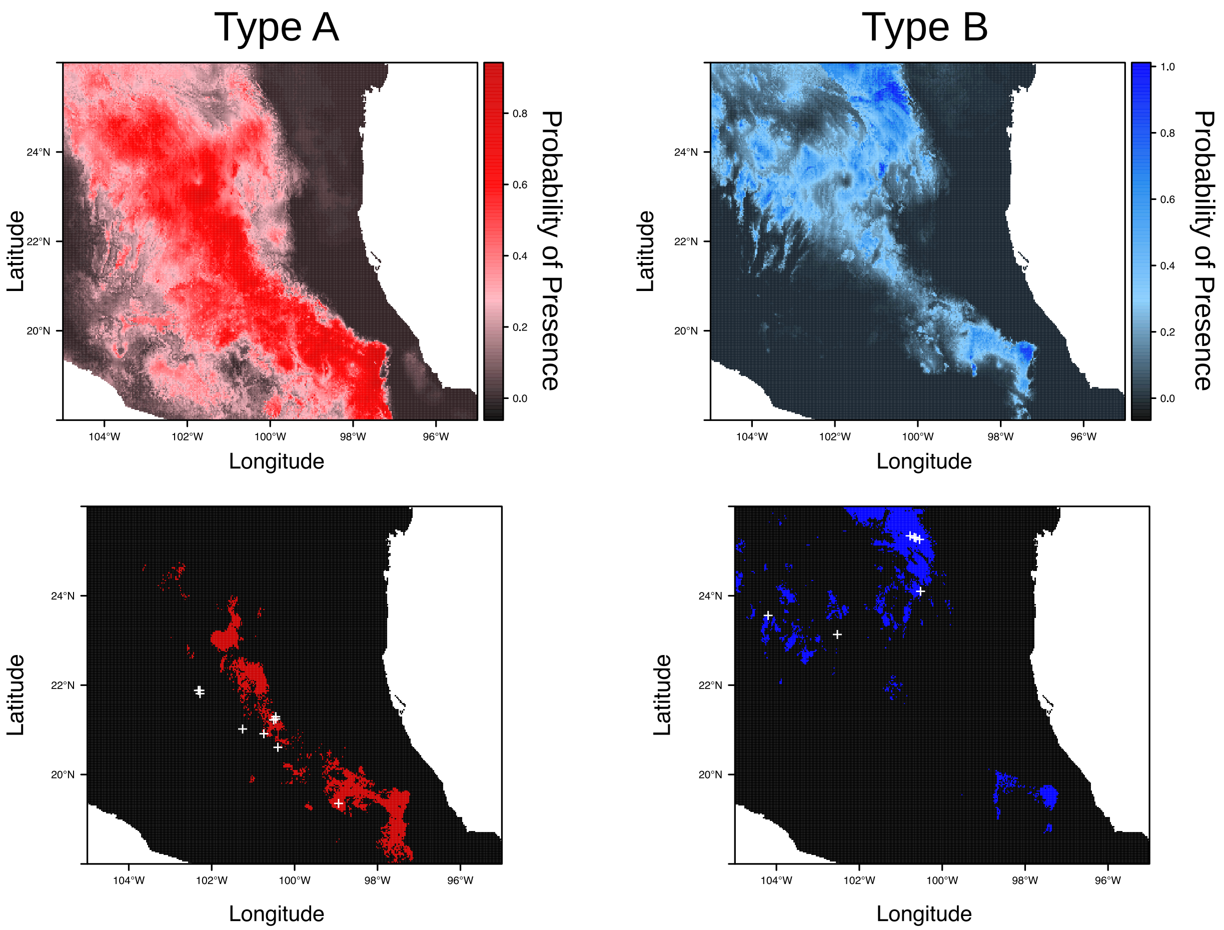

Figure S13. Ecological Niche Models generated in MAXENT[58] using 12 Type A (left) and 9 Type B (right) Mojave Rattlesnakes from the Central Mexican Plateau lineage scaled by probability of presence (pp). Lower maps display model distributions where each venom type is expected to occur based on a threshold point where model sensitivity and specificity are highest (pp $>$ 0.64 for A's, pp $>$ 0.51 for B's).

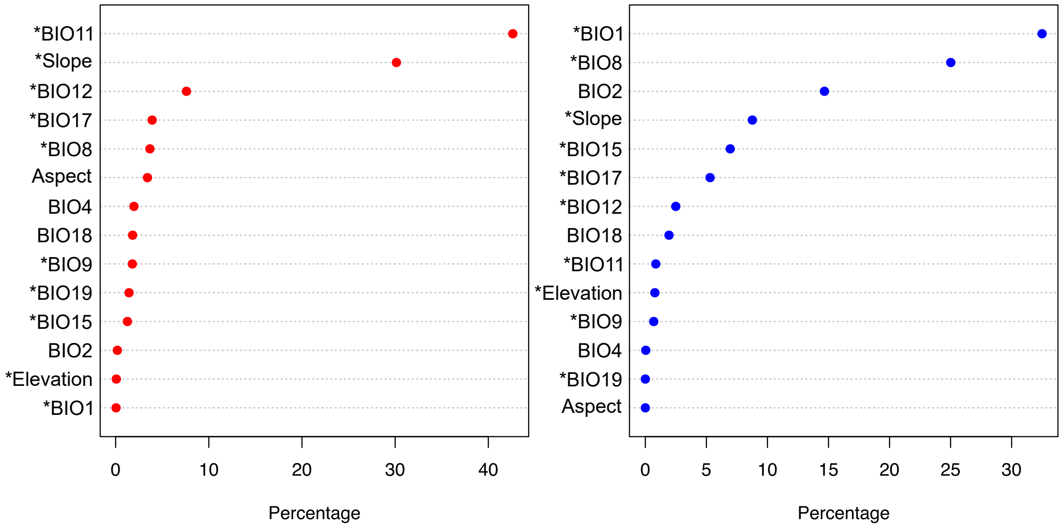

Figure S14. Model response to variables included in the Ecological Niche Models from MAXENT[58] for Type A (left) and Type B (right) venoms from the Sonoran lineage. Asterisks (*) indicate variables that were significantly different between the two models. BIO1 = Annual Mean Temperature, BIO2 = Mean Diurnal Range (Mean of monthly (max temp - min temp)), BIO4 = Temperature Seasonality (standard deviation *100), BIO8 = Mean Temperature of Wettest Quarter, BIO9 = Mean Temperature of Driest Quarter, BIO11 = Mean Temperature of Coldest Quarter, BIO12 = Annual Precipitation, BIO15 = Precipitation Seasonality (Coefficient of Variation), BIO17 = Precipitation of Driest Quarter, BIO18 = Precipitation of Warmest Quarter, BIO19 = Precipitation of Coldest Quarter.

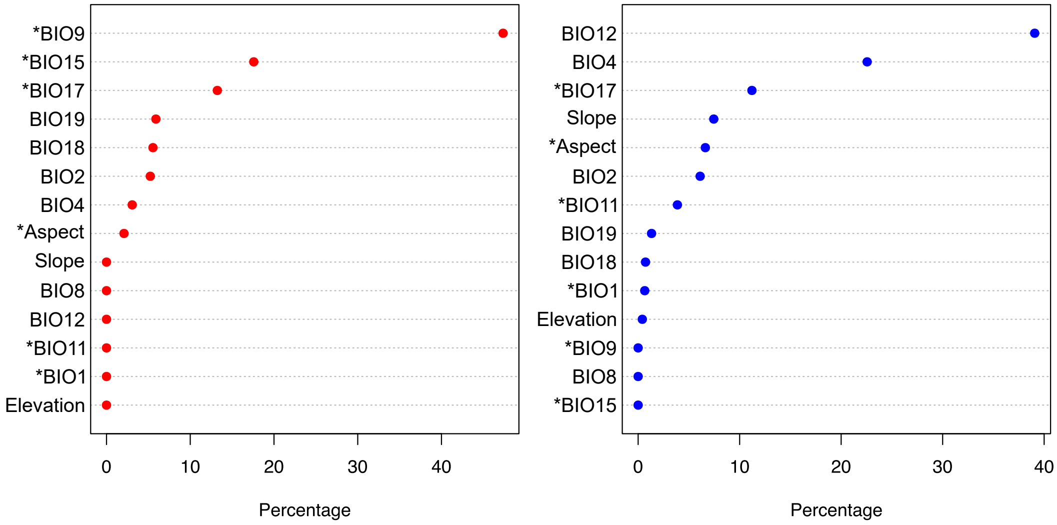

Figure S15. Model response to variables included in the Ecological Niche Models from MAXENT[58] for Type A (left) and Type B (right) venoms from the Chihuahuan lineage. Asterisks (*) indicate variables that were significantly different between the two models. BIO1 = Annual Mean Temperature, BIO2 = Mean Diurnal Range (Mean of monthly (max temp - min temp)), BIO4 = Temperature Seasonality (standard deviation *100), BIO8 = Mean Temperature of Wettest Quarter, BIO9 = Mean Temperature of Driest Quarter, BIO11 = Mean Temperature of Coldest Quarter, BIO12 = Annual Precipitation, BIO15 = Precipitation Seasonality (Coefficient of Variation), BIO17 = Precipitation of Driest Quarter, BIO18 = Precipitation of Warmest Quarter, BIO19 = Precipitation of Coldest Quarter.

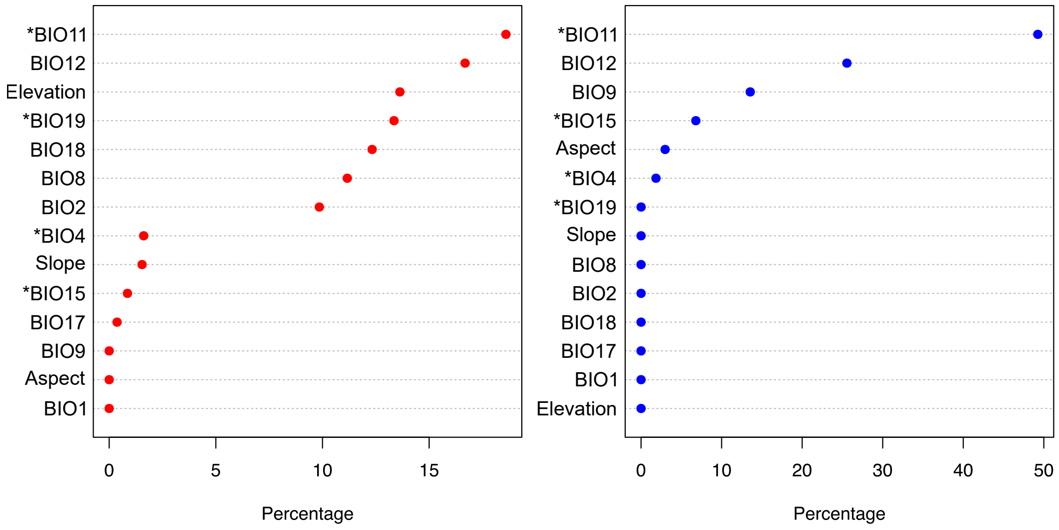

Figure S16. Model response to variables included in the Ecological Niche Models from MAXENT[58] for Type A (left) and Type B (right) venoms from the Central Mexican Plateau lineage. Asterisks (*) indicate variables that were significantly different between the two models. BIO1 = Annual Mean Temperature, BIO2 = Mean Diurnal Range (Mean of monthly (max temp - min temp)), BIO4 = Temperature Seasonality (standard deviation *100), BIO8 = Mean Temperature of Wettest Quarter, BIO9 = Mean Temperature of Driest Quarter, BIO11 = Mean Temperature of Coldest Quarter, BIO12 = Annual Precipitation, BIO15 = Precipitation Seasonality (Coefficient of Variation), BIO17 = Precipitation of Driest Quarter, BIO18 = Precipitation of Warmest Quarter, BIO19 = Precipitation of Coldest Quarter.
